# Distinct Upstream Role of Type I IFN Signaling in Hematopoietic Stem Cell-Derived and Epithelial Resident Cells for Concerted Recruitment of Ly-6C^hi^ Monocytes and NK Cells via CCL2-CCL3 Cascade

**DOI:** 10.1371/journal.ppat.1005256

**Published:** 2015-11-30

**Authors:** Erdenebileg Uyangaa, Jin Hyoung Kim, Ajit Mahadev Patil, Jin Young Choi, Seong Bum Kim, Seong Kug Eo

**Affiliations:** 1 College of Veterinary Medicine and Bio-Safety Research Institute, Chonbuk National University, Iksan, Republic of Korea; 2 Department of Bioactive Material Sciences, Graduate School, Chonbuk National University, Jeonju, Republic of Korea; University of Southern California, UNITED STATES

## Abstract

Type I interferon (IFN-I)-dependent orchestrated mobilization of innate cells in inflamed tissues is believed to play a critical role in controlling replication and CNS-invasion of herpes simplex virus (HSV). However, the crucial regulators and cell populations that are affected by IFN-I to establish the early environment of innate cells in HSV-infected mucosal tissues are largely unknown. Here, we found that IFN-I signaling promoted the differentiation of CCL2-producing Ly-6C^hi^ monocytes and IFN-γ/granzyme B-producing NK cells, whereas deficiency of IFN-I signaling induced Ly-6C^lo^ monocytes producing CXCL1 and CXCL2. More interestingly, recruitment of Ly-6C^hi^ monocytes preceded that of NK cells with the levels peaked at 24 h post-infection in IFN-I–dependent manner, which was kinetically associated with the CCL2-CCL3 cascade response. Early Ly-6C^hi^ monocyte recruitment was governed by CCL2 produced from hematopoietic stem cell (HSC)-derived leukocytes, whereas NK cell recruitment predominantly depended on CC chemokines produced by resident epithelial cells. Also, IFN-I signaling in HSC-derived leukocytes appeared to suppress Ly-6G^hi^ neutrophil recruitment to ameliorate immunopathology. Finally, tissue resident CD11b^hi^F4/80^hi^ macrophages and CD11c^hi^EpCAM^+^ dendritic cells appeared to produce initial CCL2 for migration-based self-amplification of early infiltrated Ly-6C^hi^ monocytes upon stimulation by IFN-I produced from infected epithelial cells. Ultimately, these results decipher a detailed IFN-I–dependent pathway that establishes orchestrated mobilization of Ly-6C^hi^ monocytes and NK cells through CCL2-CCL3 cascade response of HSC-derived leukocytes and epithelium-resident cells. Therefore, this cascade response of resident–to-hematopoietic–to-resident cells that drives cytokine–to-chemokine–to-cytokine production to recruit orchestrated innate cells is critical for attenuation of HSV replication in inflamed tissues.

## Author Summary

Herpes simplex virus type 1 and 2 (HSV-1 and HSV-2) are the most common cause of genital ulceration in humans worldwide with lifelong latent infection after peripheral replication in mucosal tissues. Furthermore, acquisition of human immunodeficiency virus (HIV) is increased in HSV-infected individuals, underscoring the contribution of this virus in facilitating increased susceptibility to other microbial pathogens. Therefore, it is imperative to characterize the host defense to HSV infection and identify key components that regulate virus resistance, in order to devise therapeutic strategy. Although type I interferon (IFN-I)-dependent orchestrated mobilization of innate cells in inflamed tissues is considered a key player to control replication and CNS-invasion of HSV, the regulators and cell population that are affected by IFN-I to establish the orchestrated environment of innate cells in HSV-infected tissues are largely unknown. In the present study, we demonstrate that IFN-I signal governs the sequential recruitment of Ly-6C^hi^ monocytes and then NK cells into mucosal tissues, depending on CCL2-CCL3 cascade mediated by HSC-derived leukocytes and epithelial resident cells, respectively. Also, tissue resident CD11b^hi^F4/80^hi^ macrophages and CD11c^hi^EpCAM^+^ dendritic cells were involved in producing the initial CCL2 for migration-based self-amplification of rapidly infiltrated Ly-6C^hi^ monocytes through stimulation by IFN-I produced from infected epithelial cells. This study deciphers detailed IFN-I-dependent pathway that establishes orchestrated mobilization of Ly-6C^hi^ monocytes and NK cells through CCL2-CCL3 cascade.

## Introduction

Herpes simplex virus types 1 and 2 (HSV-1 and HSV-2) are significant human pathogens and the most common cause of genital ulceration in humans worldwide [1,2]. Typically, infection with HSV-1 or -2 via genital routes results in a lifelong latent infection of the host after peripheral replication in mucosal tissues [3], thereby providing potential transmission to neighbor hosts in response to reactivation. In addition, acquisition of human immunodeficiency virus (HIV) is increased 2- to 3-fold in HSV-infected individuals, underscoring the contribution of this virus in facilitating increased susceptibility to other microbial pathogens [4–6]. Consequently, it is imperative to characterize the host defense to HSV infection and identify key components that regulate virus resistance in order to devise a strategy that will reduce viral prevalence.

The development of a mouse model of vaginal HSV infection has provided a significant contribution to our understanding of innate and adaptive immune responses to HSV infection in mucosal tissues [7,8]. In the rodent model, viral replication is initially limited to the vaginal mucosa [1–3] followed by spread into the central nervous system (CNS) upon retrograde transport of virions into the sacral ganglia, resulting in fatal paralysis [1–3]. Investigators have demonstrated the importance of the innate immune responses, including natural killer (NK), NKT cells, monocytes, and neutrophils as well as the production of type I interferons (IFN-α/β), interleukin (IL)-12, and IL-18, in suppressing viral replication and reducing virus-mediated mortality [9–13]. Also, robust IFN-γ-producing T-helper 1 (Th1) CD4^+^ T-cell immunity is required for protection against primary and secondary HSV-1 or -2 infection via the mucosal route [14–16]. Therefore, orchestrated mobilization of effector innate and adaptive immune cells (monocytes, NK, and T cells) in mucosal tissues appears to play a crucial role in providing effective protection against HSV infection without detrimental pathology.

NK cells are known to play a critical role in HSV control by recognizing and killing infected cells upon engagement of NK cell-activating receptors by altered self or viral ligand pairs expressed by infected cells [17–19]. These antiviral NK cells are thought to be regulated by type I IFN (IFN-I) signals through promotion of cell entry into the cell cycle, proliferation, survival, and cytotoxicity [20,21]. Some of these effects may be indirectly mediated by IL-15, although this is debatable [20,21]. Also, the coordinated roles of CD11b^+^Ly-6C^hi^ monocytes in various immunopathogeneses caused by pathogenic infection were recently described [22,23]. After infection, CD11b^+^Ly-6C^hi^ monocytes emigrate from the bone marrow (BM) into the bloodstream through CCR2-receptor–mediated signaling and differentiate into dendritic cells (DCs) that produce tumor necrosis factor (TNF)-α and nitric oxide (NO) [24,25]. These cells, known as Tip-DCs, are required for lysis and clearance of pathogens such as *Listeria monocytogenes* [25] and *Toxoplasma gondii* [26]. Furthermore, monocyte-derived DCs appear to migrate from the inflamed tissues to the draining lymph node (DLN) and prime naïve CD4^+^ T cells in *Leishmania major* infection [27]. Likewise, the chemokine CCL2 recruits Ly-6C^hi^ monocytes to mucosal tissues after HSV infection, where they appear to exert tailored protective immunity by secreting antimicrobial factors (TNF-α, NO) and stimulating antiviral Th1 immunity through differentiation into inflammatory DCs [28]. In accordance with these findings, IFN-I have been shown to regulate CD11b^+^Ly-6C^hi^ monocyte recruitment during viral infection [29,30], as well as during chronic inflammation [31], by inducing CCL2. Therefore, defining the role of IFN-I signaling in the differentiation and recruitment of monocytes is required to better understand inflammatory responses for the development of therapeutic strategies. However, the crucial regulatory factors and the cell populations that are affected by IFN-I to establish the orchestrated mobilization of innate immune cells such as NK cells and CD11b^+^Ly-6C^hi^ monocytes in mucosal tissues following mucosal HSV infection are largely unknown.

Therefore, the experiments presented here were undertaken (a) to define the role of IFN-I in the regulation of innate immune cell populations such as monocytes and NK cells, and (b) to identify specific cell populations and molecular regulators affected by IFN-I that are involved in establishing early orchestrated mobilization of innate cells in mucosal tissues following mucosal HSV infection. Somewhat intriguingly, our data revealed that IFN-I signaling regulated the sequential recruitment of CD11b^+^Ly-6C^hi^ monocytes followed by NK cells, which was closely associated with the cascade response of CCL2 and CCL3 production in mucosal tissues; i.e., CD11b^+^Ly-6C^hi^ monocyte infiltration peaked at around 24 h after infection, whereas NK cell infiltration peaked at 48 h post-infection (pi). Furthermore, early recruitment of CD11b^+^Ly-6C^hi^ monocytes was dominantly regulated by CCL2 produced from hematopoietic stem cell (HSC)-derived leukocytes, whereas NK cell recruitment depended on CCL3 and other CC chemokines produced from resident epithelium cells. Also, early CCL2 proteins secreted from tissue resident CD11b^hi^F4/80^hi^ macrophages and CD11c^hi^EpCAM^+^ DCs upon stimulation of IFN-I produced from infected epithelium were required for migration-based self-amplification of CD11b^+^Ly-6C^hi^ monocytes in mucosal tissues. Taken together, these results provide new insights into the role of IFN-I signaling by defining a unique cascade pathway in the regulation of chemokine responses required for the early orchestrated mobilization of innate immune cells.

## Results

### IFN-I signaling is involved in the rapid and concerted recruitment of Ly-6C^hi^ monocytes and CD11c^+^ DCs

IFN-I signaling plays an important role in conferring protection against HSV-1 mucosal infection [18], as confirmed by our preliminary results (S1A and S1B Fig). Also, IFN-I signaling was required to control shedding of infectious virus (S1C Fig) and to prevent CNS invasion of HSV-1 from mucosal tissues (S1D–S1H Fig). Furthermore, it seemed likely that IFN-I signaling played a crucial role in establishing an early orchestrated environment of cytokine and chemokine expression for viral clearance (S2 Fig). Notably, BL/6 mice showed markedly enhanced expression of type I IFNs (IFN-α/β), CCL2, CCL3, and CCR5 (involved in recruitment of monocytes and NK cells), and CXCL10 (involved in recruitment of Th1 CD4^+^ T cells) in the vaginal tract, compared to *IFNAR* KO mice.

**Fig 1:**
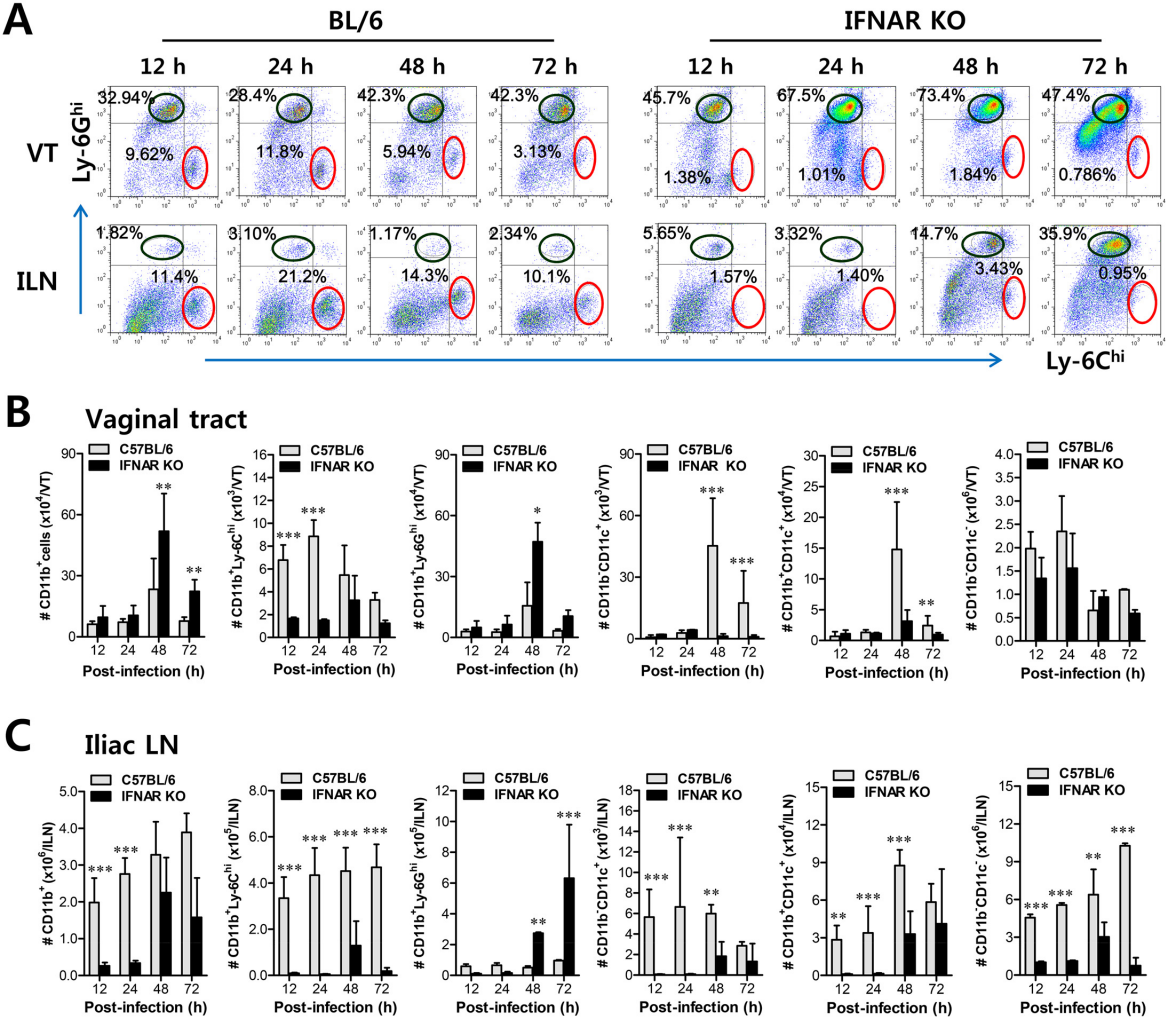
IFN-I signaling is essential to induce the rapid and concerted recruitment of Ly-6C^hi^ monocytes and CD11c^+^ DCs in mucosal and draining LN tissues. **(A)** Leukocyte infiltration of vaginal tract and iliac LN in infected BL/6 or *IFNAR* KO mice. **(B,C)** Accumulated number of infiltrated leukocyte subsets. Cells were prepared from vaginal tract (B; VT) and iliac LN (C; ILN) by collagenase digestion at 12, 24, 48, and 72 h pi and subcellular proportions of CD11b^+^Ly-6C^hi^ and CD11b^+^Ly-6G^hi^ leukocytes were determined using flow cytometric analysis. Values in the representative dot-plots denote the average percentages of each population derived from at least four independent samples after gating on CD11b^+^ cells. Data in the bar chart represent the average ± SD of values derived from three individual experiments (*n* = 4–5). *, *p*<0.05; **, *p*<0.01; ***, *p*<0.001 compared with the levels of BL/6 or *IFNAR* KO mice at the indicated time point.

However, the crucial regulatory factors that establish the early orchestrated environment of infiltrated leukocytes in mucosal tissues and their cellular source have been not defined in depth. CD11b^+^Ly-6C^hi^ monocytes play a pivotal role in exerting direct antimicrobial activity or further differentiate into inflammatory DCs, which participate in innate and adaptive protective immunity [24,25]. In addition, since our preliminary data showed that expression of the chemokine CCL2, a major factor in monocyte recruitment, was early and markedly enhanced in the vaginal tract of WT BL/6 mice upon mucosal HSV-1 infection (S2 Fig), we performed kinetic examinations of recruited myeloid-derived leukocyte subpopulations in the vaginal tract and its draining LN (iliac LN) 12, 24, 48, and 72 h after infection. IFN-I signal was found to be critical for recruitment of CD11b^+^Ly-6C^hi^ monocytes in vaginal tract and iliac LN during the early phase of HSV-1 mucosal infection (Fig 1A). Notably, WT BL/6 mice showed early infiltration of CD11b^+^Ly-6C^hi^ monocytes with a 10- to 20-fold increase in the vaginal tract and iliac LN at 24 h after infection, compared to *IFNAR* KO mice. In contrast, *IFNAR* KO mice contained a greater number of accumulated CD11b^+^Ly-6G^hi^ granulocytes in the vaginal tract with levels peaking at 48 h pi, and the number of iliac CD11b^+^Ly-6G^hi^ granulocytes increased up to 72 h pi. These results indicate that maximal infiltration of CD11b^+^Ly-6C^hi^ monocytes in the vaginal tract of BL/6 mice is established at around 24 h after infection, whereas the infiltration of CD11b^+^Ly-6G^hi^ granulocytes in *IFNAR* KO mice peaked at 48 h pi. When total accumulated subpopulations of myeloid-derived leukocytes and DCs were examined during the early phase after HSV-1 infection (12, 24, 48, and 72 h pi), maximum accumulation of CD11b^+^Ly-6C^hi^ monocytes was detected in the vaginal tract of BL/6 mice with 4- to 5-fold increased levels at 24 h pi, despite a moderately increased number of total CD11b^+^ myeloid-derived cells in *IFNAR KO* mice (Fig 1B). In contrast, the total number of CD11^+^ DCs and its myeloid DC subpopulation (CD11b^+^CD11c^+^) was increased in the vaginal tract of BL/6 mice, with a delayed peak at 48 h pi compared to *IFNAR* KO mice. Likewise, iliac LN of BL/6 mice also showed early increased levels of total CD11b^+^Ly-6C^hi^ monocytes with saturated levels at 24 h pi, and levels of CD11c^+^ DCs, the myeloid DC subset (CD11c^+^CD11b^+^) and other DC subsets (CD11b^−^CD11c^+^) peaked at around 48 h pi, whereas in *IFNAR* KO mice CD11b^+^Ly-6G^hi^ granulocytes showed gradually increasing levels up to 72 h pi (Fig 1C). To summarize, interestingly, these results indicate that infiltration of CD11b^+^Ly-6C^hi^ monocytes (levels of which peaked at 24 h pi) precede that of CD11c^+^DCs (levels of which peaked at 48 h) in mucosal tissues upon HSV-1 infection. Therefore, these results suggest that IFN-I signaling plays a crucial role in establishing early infiltration of CD11b^+^Ly-6C^hi^ monocytes and CD11c^+^ DCs in the submucosa area of the vaginal tract with a concerted pattern of recruitment, which could subsequently provide a well-controlled inflammatory reaction and adaptive protective immunity.

### IFN-I signal differentially induces functional activation of Ly-6C^hi^ and Ly-6C^lo^ monocytes

**Fig 2:**
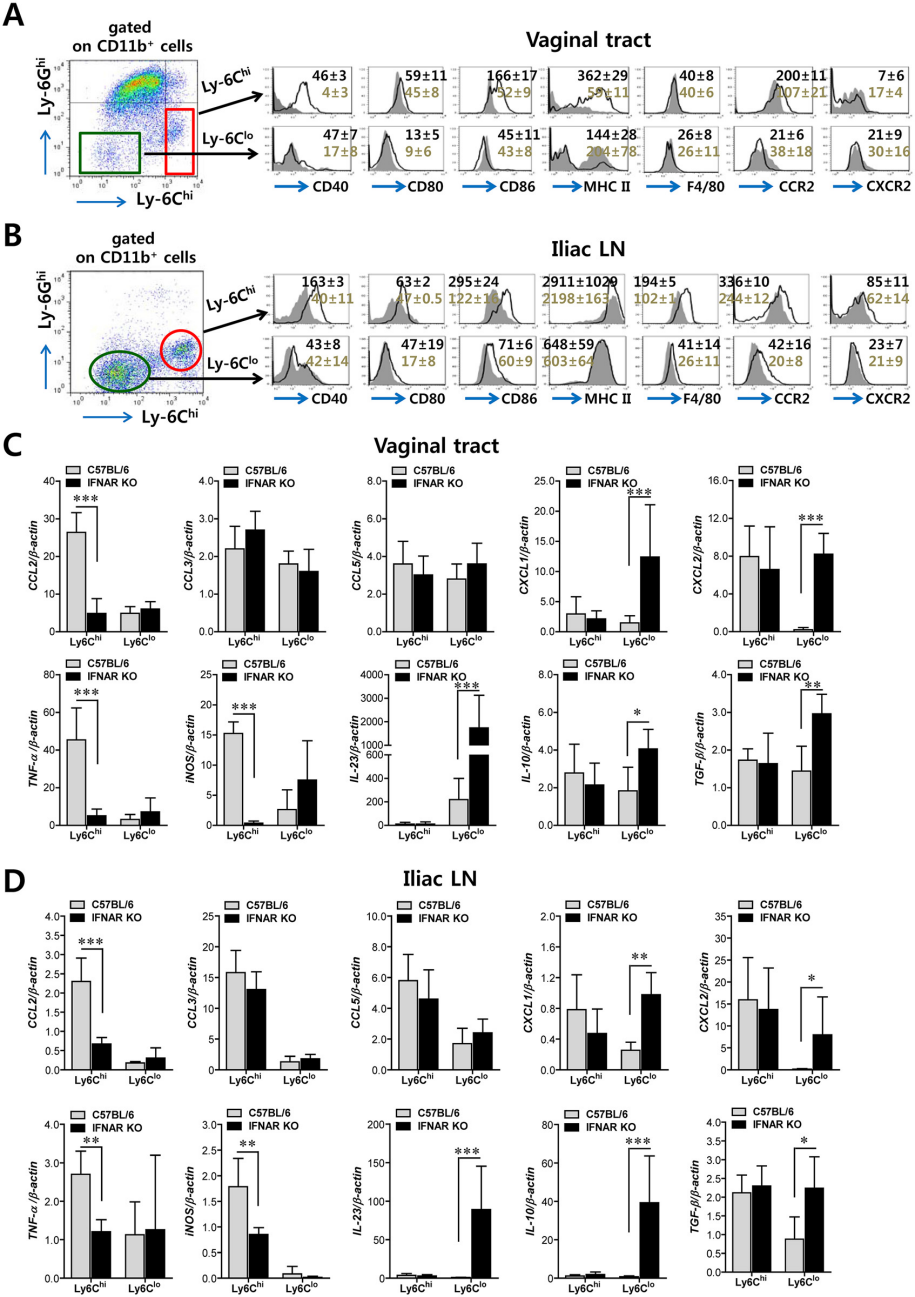
Differentiation levels and cytokine expression profile of infiltrated CD11b^+^Ly-6C^hi^ and CD11b^+^Ly-6C^lo^ monocytes in vaginal tract and iliac LN. **(A,B)** Differentiation levels of CD11b^+^Ly-6C^hi^ and CD11b^+^Ly-6C^lo^ monocytes in vaginal tract and iliac LN. The expression of differentiation markers was determined by flow cytometric analysis using cells prepared from vaginal tract (A) and iliac LN (B) by collagenase digestion at 24 h pi. **(C,D)** Cytokine expression profile of sorted CD11b^+^Ly-6C^hi^ and CD11b^+^Ly-6C^lo^ monocytes. The levels of cytokine and chemokine mRNAs were determined by real-time qRT-PCR using total RNA extracted from CD11b^+^Ly-6C^hi^ and CD11b^+^Ly-6C^lo^ monocytes sorted from vaginal tract (C) and iliac LN (D) of infected BL/6 or *IFNAR* KO mice at 24 h pi. Data denote the average ± SD of values derived from three individual experiments (*n* = 5–6). *, *p*<0.05; **, *p*<0.01; ***, *p*<0.001 for comparison between the indicated groups.

In the context of inflammation, CD11b^+^Ly-6C^hi^ monocytes differentiate into inflammatory DCs that could exert tailored protective immunity [24–28]. Therefore, we were interested in testing whether CD11b^+^Ly-6C^hi^ monocytes that infiltrated into vaginal and iliac LN tissues also have the ability to present Ag as professional antigen-presenting cells similar to DCs during mucosal inflammation caused by HSV-1 infection. To this end, we further characterized CD11b^+^Ly-6C^hi^ and CD11b^+^Ly-6C^lo^ monocytes for antigen-presenting molecules and activation markers. CD11b^+^Ly-6C^hi^ monocytes from the vaginal tract of both WT BL/6 and *IFNAR* KO mice expressed higher levels of Ag presenting-related molecules (CD40, CD80, CD86, and MHC II) than CD11b^+^Ly-6C^lo^ monocytes, and these molecules showed significantly higher expression levels in CD11b^+^Ly-6C^hi^ monocytes derived from WT BL/6 mice compared to those from *IFNAR* KO mice (Fig 2A). Also, CD11b^+^Ly-6C^hi^ monocytes derived from vaginal tract of BL/6 mice displayed upregulated expression of CCR2, the receptor of CCL2. Similarly, CD11b^+^Ly-6C^hi^ monocytes in iliac LN of BL/6 mice also showed upregulated expression levels of Ag-presenting–related molecules and activation markers compared to those of *IFNAR* KO mice (Fig 2B). Since CD11b^+^Ly-6C^hi^ monocytes also exert antimicrobial activity by secreting antimicrobial factors (TNF-α, NO), we next examined the expression of cytokines and chemokines in CD11b^+^Ly-6C^hi^ and CD11b^+^Ly-6C^lo^ monocytes derived from vaginal and iliac LN tissues. Intriguingly, CD11b^+^Ly-6C^hi^ and CD11b^+^Ly-6C^lo^ monocytes showed contrasting expression patterns of cytokines and chemokines in WT BL/6 and *IFNAR* KO mice (Fig 2C). CD11b^+^Ly-6C^hi^ monocytes derived from vaginal tract of WT BL/6 mice showed markedly higher expression of TNF-α and iNOS cytokines (the main factors of TipDC) and the CCL2 chemokine (the ligand of CCR2 as a major monocyte chemotactic factor) than those of *IFNAR* KO mice. In contrast, CD11b^+^Ly-6C^lo^ monocytes from vaginal tract of *IFNAR* KO mice expressed high mRNA levels of cytokines IL-23, IL-10, and TGF-β and CXC chemokines (CXCL1, CXCL2). Similarly, iliac CD11b^+^Ly-6C^hi^ monocytes derived from WT BL/6 mice showed upregulated expression of CCL2, TNF-α, and iNOS, whereas the expression of CXC chemokines (CXCL1 and CXCL2) and cytokines (IL-23, IL-10, and TGF-β) was increased in iliac CD11b^+^Ly-6C^lo^ monocytes of *IFNAR* KO mice (Fig 2D). Collectively, these results indicate that IFN-I signal is crucial for determining the characteristics of CD11b^+^Ly-6C^hi^ and CD11b^+^Ly-6C^lo^ monocytes. Also, somewhat surprisingly, IFN-I signaling differentially affected the functional maturation of CD11b^+^Ly-6C^hi^ and CD11b^+^Ly-6C^lo^ monocytes during mucosal HSV-1-induced inflammation.

### Essential role of IFN-I signal in concerted recruitment of NK cells to mucosal tissues and their activation

**Fig 3:**
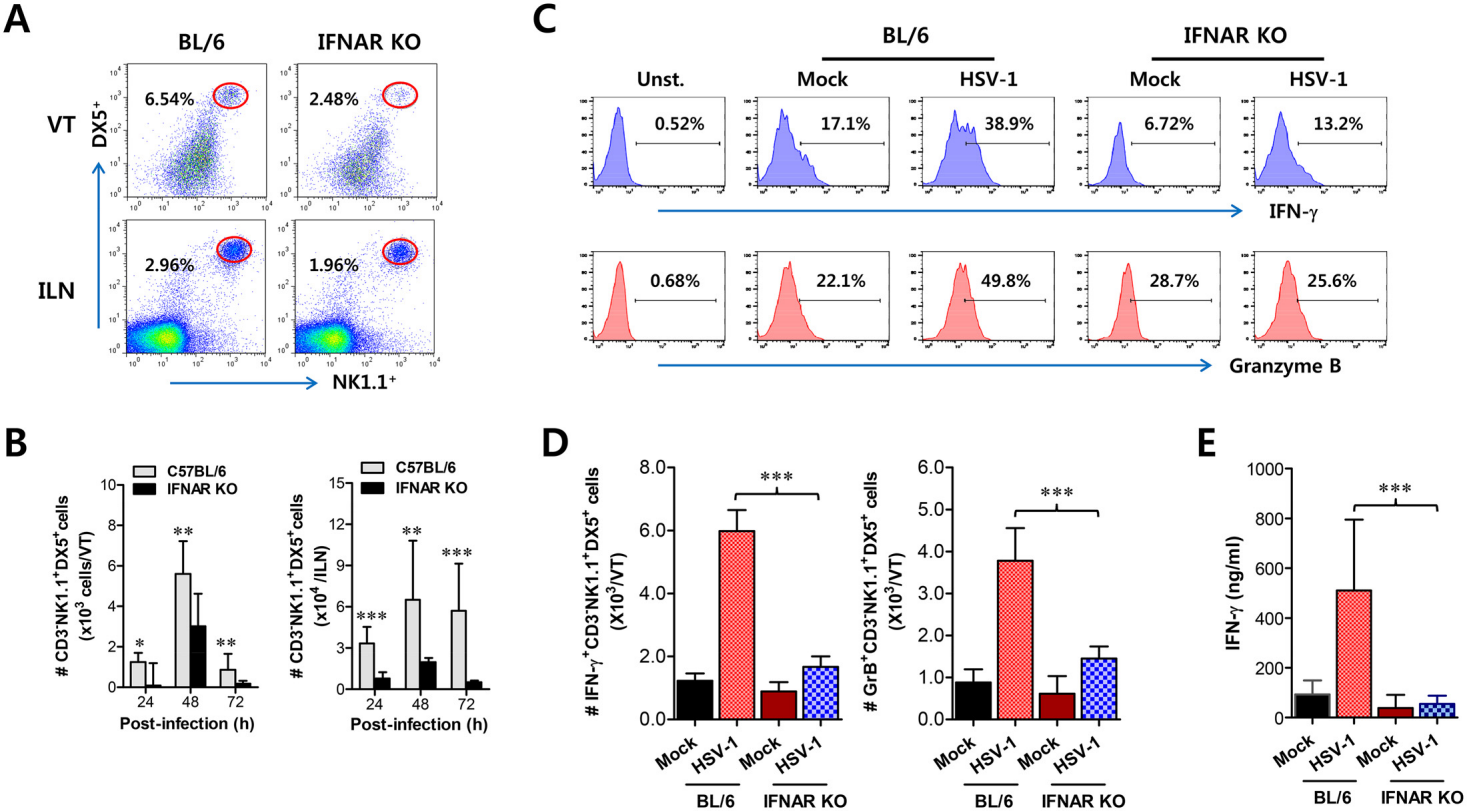
IFN-I signaling is involved in connected recruitment and activation of NK cells to CD11b^+^Ly-6C^hi^ monocytes. **(A)** NK cell infiltration of infected BL/6 or *IFNAR* KO mice. **(B)** Total accumulated NK cell number. Cells were prepared from vaginal tract (VT) and iliac LN (ILN) by collagenase digestion at 24, 48, and 72 h pi and used for analysis of NK cells. Values in dot-plots denote the average percentages of NK1.1^+^DX5^+^ NK cells derived from four independent samples after gating on CD3-negative cells at 48 h pi. **(C)** Frequency of IFN-γ or granzyme B-producing cells among NK cells. The production of IFN-γ and granzyme B by CD3^−^NK1.1^+^DX5^+^ NK cells was determined by intracellular staining after stimulation of vaginal NK cells with PMA plus ionomycin at 48 h pi. Values in histograms represent the average percentages of IFN-γ or granzyme B-producing cells among CD3^−^NK1.1^+^DX5^+^ NK cells. **(D)** Absolute number of IFN-γ or granzyme B-producing NK cells. Total number of IFN-γ or granzyme B-producing CD3^−^NK1.1^+^DX5^+^ NK cells in vaginal tract was enumerated by flow cytometric analysis using intracellular and surface staining at 48 h pi. **(E)** Secreted IFN-γ levels in vaginal tract. Secreted IFN-γ levels were determined by ELISA at 48 h pi using vaginal lavages. Data in bar chart represent the average ± SD of values derived from three individual experiments (*n* = 4–5). **, *p*<0.01; ***, *p*<0.001 compared with the levels of *IFNAR* KO mice.

Together with CD11b^+^Ly-6C^hi^ monocytes, NK cells are a critical innate cell population for providing protection against mucosal HSV-1 infection via production of IFN-γ [17–19]. Since CD11b^+^Ly-6C^hi^ monocytes and CD11c^+^ DCs were shown to be consecutively recruited to the vaginal tract, we were also interested in the recruitment kinetics and activation of NK cells in IFNAR-ablated mice. Before virus infection, the frequency and number of CD3^−^NK1.1^+^DX5^+^ NK cells were comparable in vaginal tract and iliac LN of both BL/6 and *IFNAR* KO mice, except liver tissue (S3 Fig), which indicates that IFN-I signaling may not contribute to basal recruitment of NK cells in primary target tissues of HSV-1 mucosal infection. However, it was revealed that IFN-I signaling plays a crucial role in recruiting CD3^−^NK1.1^+^DX5^+^ NK cells into vaginal tract after HSV-1 infection. Notably, WT BL/6 mice displayed delayed recruitment of CD3^−^NK1.1^+^DX5^+^ NK cells in the vaginal tract with the levels peaking at 48 h pi, compared to CD11b^+^Ly-6C^hi^ monocytes, and an increased frequency of NK cells, compared to *IFNAR* KO mice (Fig 3A). Also, iliac NK cell frequency was moderately increased in WT BL/6 mice compared to *IFNAR* KO mice. Supporting these findings, total accumulated number of CD3-NK1.1^+^DX5^+^ NK cells in vaginal tract and iliac LN was greater in WT BL/6 mice, with levels peaking at 48 h pi, than in *IFNAR* KO mice (Fig 3B). In addition, when the activation levels of NK cells were examined by analysis of NK cells producing IFN-γ and granzyme B, the ablation of IFN-I signal resulted in reduced production of IFN-γ and granzyme B from CD3^−^NK1.1^+^DX5^+^ NK cells (Fig 3C), and a decrease in the total number of NK cells producing IFN-γ or granzyme B (Fig 3D). Consistent with these findings, lower levels of IFN-γ protein in vaginal lavages of IFNAR KO mice were detected at 48 h pi compared to WT BL/6 mice (Fig 3E). Collectively, these results indicate that NK cell recruitment in vaginal tract follows that of CD11b^+^Ly-6C^hi^ monocytes like CD11c^+^ DCs, and that IFN-I signal also has a critical contribution to the early concerted recruitment and activation of CD11b^+^Ly-6C^hi^ monocytes and NK cells in mucosal tissues following HSV-1 infection.

### Cascade responses of CCL2-CCL3 are kinetically associated with concerted recruitment of Ly-6C^hi^ monocytes and NK cells

**Fig 4:**
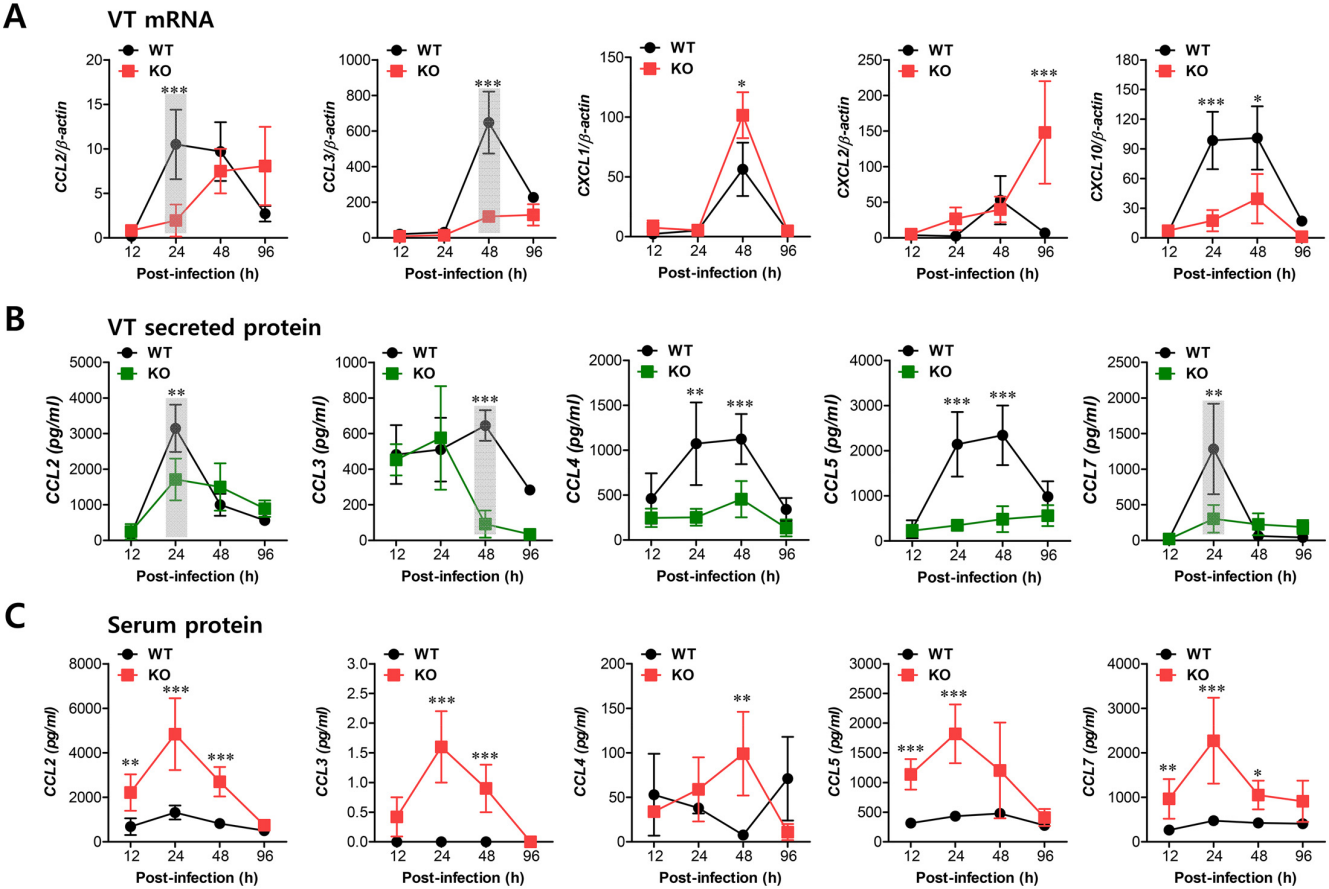
Sequential responses of chemokines in response to mucosal infection with HSV-1. **(A)** Expression of chemokine mRNA in vaginal tract of BL/6 or *IFNAR* KO mice following mucosal infection with HSV-1. The levels of chemokine mRNAs were determined by real-time qRT-PCR using total RNA extracted from vaginal tract (VT) tissues at 12, 24, 48, and 96 h pi. **(B)** Secreted levels of chemokine proteins in vaginal tract. **(C)** Serum chemokine protein levels. The levels of chemokine proteins were determined by CBA using vaginal lavages and sera at 12, 24, 48, and 96 h pi. Data show the average ± SD of values derived from three individual experiments (*n* = 5–6). Areas highlighted by gray color denote interesting points. *, *p*<0.05; **, *p*<0.01; ***, *p*<0.001 compared with the levels in *IFNAR* KO mice at the indicated time point.

Sequential responses of chemokines appear to be necessary for the selective and tailored environment of infiltrated leukocytes that provides effective protection against mucosal HSV-1 infection, even though the chemokine responses can be redundant [32,33]. Moreover, our data demonstrated that infiltration of CD11b^+^Ly-6C^hi^ monocytes preceded that of NK cells during mucosal inflammation; CD11b^+^Ly-6C^hi^ monocyte infiltration peaked at around 24 h pi, whereas NK cell infiltration peaked at 48 h after mucosal HSV-1 infection. Therefore, we were interested in the regulatory molecules that are involved in establishing this tailored environment of sequential CD11b^+^Ly-6C^hi^ monocyte and NK cell infiltration in vaginal tissues. To this end, we performed a kinetic examination of the expression and secretion of several CC and CXC chemokines in vaginal tissues and lavages at different time points up to 96 h pi. Somewhat intriguingly, our data revealed that CCL2 and CCL3 expression was evident early, with cascade responses peaking at subsequent time points. Hence, expression of CCL2, a ligand of CCR2, began as early as 12 h pi in vaginal tract of WT BL/6 mice and increased sharply in a time-dependent manner (Fig 4A). Maximal levels of vaginal CCL2 induction in WT mice were observed at around 24 h pi and had declined 80% by 96 h pi, whereas *IFNAR* KO mice showed a delayed and lower expression of vaginal CCL2 with levels gradually increasing after 48 h pi. Vaginal expression of CCL3, a ligand of CCR1 and CCR5, peaked at 48 h pi in WT mice, whereas *IFNAR* KO mice showed a drastically decreased expression level of CCL3. Other CXC chemokines (CXCL1, CXCL2) that are involved in recruitment of neutrophils via CXCR2 showed higher expression levels in the vaginal tract of *IFNAR* KO mice compared to WT BL/6 mice. Notably, CXCL2 expression gradually increased in *IFNAR* KO mice after 48 h pi, whereas WT BL/6 mice showed decreased levels at 96 h pi. Regarding secreted chemokine proteins in vaginal lavages, ligands of CCR2 (CCL2, CCL7) were secreted at a higher level in WT BL/6 mice with reaching a maximum level at 24 h pi, compared to secretion in *IFNAR* KO mice (Fig 4B). In contrast, ligands of CCR5 (CCL3, CCL4, CCL5) showed delayed peaks at around 48 h pi and such levels were markedly higher in WT mice than in *IFNAR* KO mice. These results indicate that cascade responses of CC chemokines, especially CCL2 and CCL3, appear to be closely associated with consecutive recruitment of CD11b^+^Ly-6C^hi^ monocytes and NK cells in the vaginal tract of WT BL/6 mice, whereas enhanced induction of CXC chemokine (CXCL1, CXCL2) in *IFNAR* KO mice appears to be involved in neutrophil recruitment in the vaginal tract. However, systemic levels of most chemokine proteins were markedly higher in the sera of *IFNAR* KO mice compared to WT BL/6 mice (Fig 4C). Although cellular sources for huge amount of chemokine secretion in sera of *IFNAR* KO mice were not defined, it is likely that various cell types derived from *IFNAR* KO mice, including epithelial cells, fibroblast, and endothelial cells, are highly susceptible to HSV-1 replication, thereby secreting chemokines in sera by severe inflammation in whole body. In addition, this result suggests that enhanced chemokine levels in the serum may cause disturbed trafficking of various leukocytes in *IFNAR* KO mice.

### IFN-I signal on leukocytes derived from HSC lineage is required for normal recruitment of Ly-6C^hi^ monocytes, but not NK cells

**Fig 5:**
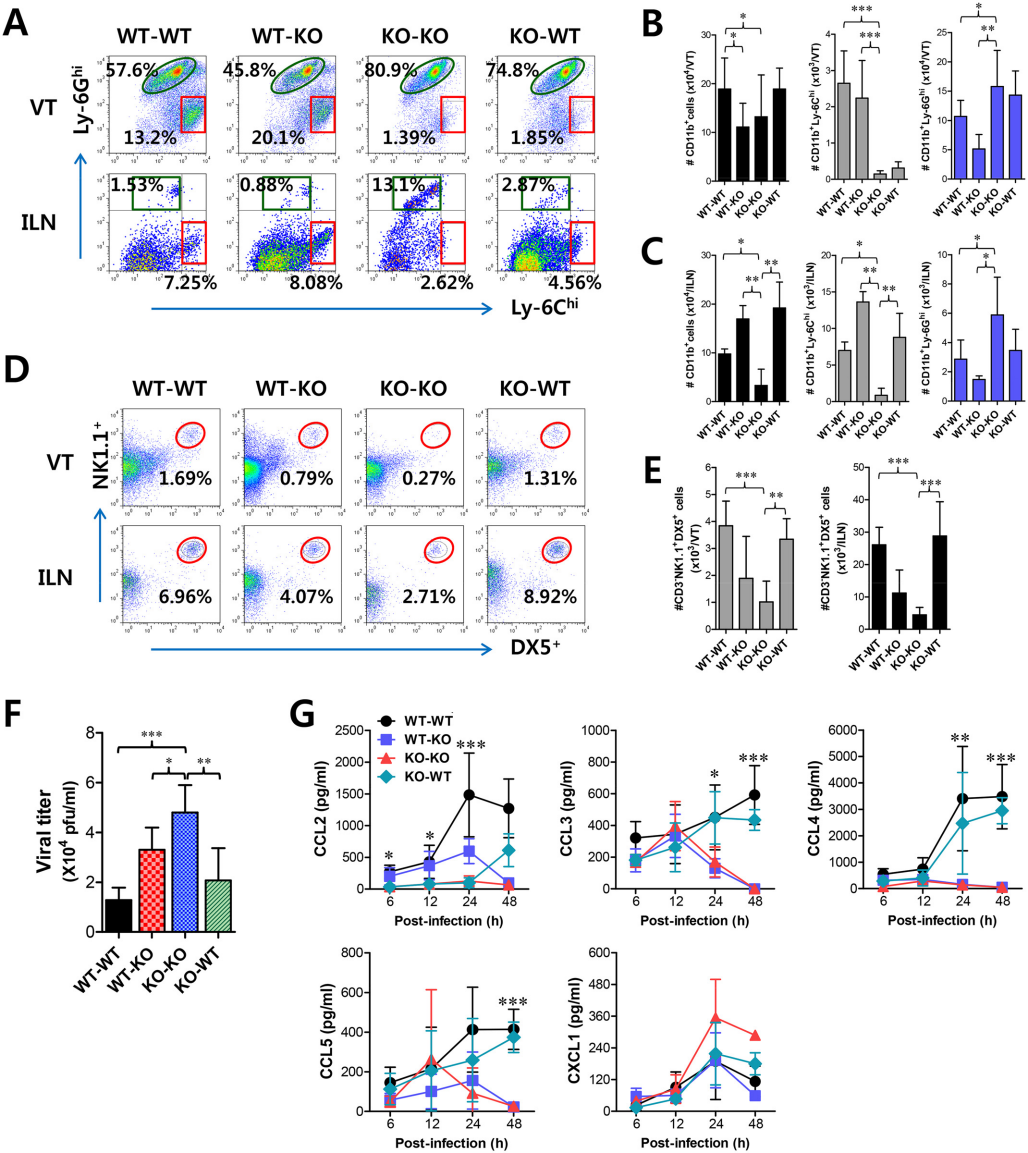
IFN-I signaling on infiltrated leukocytes derived from HSC lineage is required for normal recruitment of CD11b^+^Ly-6C^hi^ monocytes, but not NK cells. BM cells from BL/6 (WT) or *IFNAR* KO (KO) were grafted into lethally irradiated BL/6 or *IFNAR* KO recipient mice, which were infected i.vag. with HSV-1. **(A)** Leukocyte infiltration in vaginal tract and iliac LN of infected recipients. **(B,C)** Accumulated number of infiltrated leukocyte subsets. Cells were prepared from vaginal tract (B; VT) and iliac LN (C; ILN) by collagenase digestion at 24 h pi and subcellular proportions of CD11b^+^Ly-6C^hi^ and CD11b^+^Ly-6G^hi^ leukocytes were determined using flow cytometric analysis. **(D,E)** Frequency and absolute number of infiltrated NK cells in VT and ILN of the recipients. Cells prepared from VT and ILN of the recipients were used to analyze the frequency (D) and total number (E) of CD3^−^NK1.1^+^DX5^+^ NK cells at 48 h pi. **(F)** Viral titers in vaginal lavages. Viral titers in vaginal lavages collected at 48 h pi were determined by plaque assay. **(G)** Chemokine secretion in vaginal lavages of the recipients. The levels of chemokine proteins were determined by CBA using vaginal lavages at 6, 12, 24, and 48 h pi. Values in dot-plots represent the average percentages of each population derived from four independent samples, and data in graphs represent the average ± SD of values derived from three individual experiments (*n* = 4–5). *, *p*<0.05; **, *p*<0.01; ***, *p*<0.001 compared with the level of the indicated group.

Since CD11b^+^Ly-6C^hi^ monocytes and NK cells showed concerted recruitment with levels peaking at different time points associated with the cascade response of CCL2-CCL3 in the vaginal tract, we were interested in dissecting the contribution of resident (stromal and epithelial cells) and HSC-derived leukocytes in this process. To achieve this, we used a BM chimeric model of WT BL/6 and *IFNAR* KO mice. As expected, *IFNAR* KO recipients of *IFNAR* KO BM donor cells (KO-KO chimera) showed a diminished frequency of CD11b^+^Ly-6C^hi^ monocytes in vaginal and iliac LN, compared to WT recipients of WT BM donor cells (WT-WT chimera) (Fig 5A). Also, somewhat intriguingly, *IFNAR* KO recipients of WT BM donor cells (WT-KO chimeras) showed comparable recruitment of CD11b^+^Ly-6C^hi^ monocytes in the vaginal tract and iliac LN to WT-WT chimeras, but WT BL/6 recipients of *IFNAR* KO BM donor cells (KO-WT chimeras) contained a lower frequency of CD11b^+^Ly-6C^hi^ monocytes with levels comparable to those of KO-KO chimeras, indicating that IFN-I receptors of some leukocytes derived from HSC lineage are essential for the recruitment of CD11b^+^Ly-6C^hi^ monocytes in vaginal and iliac LN tissues. When the absolute number of recruited CD11b^+^Ly-6C^hi^ monocytes was determined, the essential role of IFN-I receptor on leukocytes derived from the HSC lineage in vaginal CD11b^+^Ly-6C^hi^ monocyte recruitment was revealed again, even though KO-WT chimeras showed slightly higher levels of total recruited CD11b^+^ cells compared to the other chimeras (Fig 5B). In contrast, IFN-I receptors in HSC-derived leukocytes appeared to be dispensable for the recruitment of CD11b^+^Ly-6G^hi^ neutrophils in the vaginal tract, because WT-KO chimeras showed a reduced number of neutrophils compared to KO-KO chimeras. Also, considerable recruitment of iliac CD11b^+^Ly-6C^hi^ monocytes in both WT-KO and KO-WT chimeras highlighted the importance of IFN-I receptors in both HSC-derived and resident cells for lymphoid tissues (Fig 5C). Therefore, these results indicate that IFN-I signal in some leukocytes derived from the HSC lineage plays a crucial role in early recruitment of CD11b^+^Ly-6C^hi^ monocytes in the vaginal tract with relatively less contribution to recruitment of CD11b^+^Ly-6C^hi^ monocytes in draining LN, even though specific leukocytes derived from the HSC lineage to involve in the recruitment of Ly-6C^hi^ monocytes were not defined. Another intriguing result is that, unlike vaginal CD11b^+^Ly-6C^hi^ monocyte recruitment, the recruitment of NK cells in the vaginal tract and iliac LN obviously depended dominantly on IFN-I receptor in resident cells; hence, KO-WT chimera showed completely recovered recruitment of CD3^−^NK1.1^+^DX5^+^ NK cells in vaginal tract and iliac LN, compared to diminished recruitment of NK cells in KO-KO and WT-KO chimeras (Fig 5D and 5E). In addition, it was likely that recovered recruitment of CD11b^+^Ly-6C^hi^ monocytes and NK cells in WT-KO and KO-WT chimeras contributed to the control of HSV-1 replication in the vaginal tract (Fig 5F). Collectively, these results suggest that IFN-I signaling in resident cells is dominantly responsible for recruiting NK cells in inflammatory mucosal tissues, in contrast to CD11b^+^Ly-6C^hi^ monocytes that depend on the IFN-I signal in BM-derived HSCs.

To further characterize the distinct dependence of vaginal recruitment of CD11b^+^Ly-6C^hi^ monocytes and NK cells on IFN-I signal, we examined cascade responses of CC and CXC chemokines in vaginal lavages obtained from four BM chimeric models following mucosal HSV-1 infection. CCL2 secretion was gradually increased in WT-KO chimera with levels detectable at 6 h, increasing at 24 h pi, and then sharply decreasing at 48 h pi (Fig 5G). However, KO-WT chimeras displayed delayed secretion of CCL2 starting at 24 h pi and gradually increasing to 48 h pi. These data indicate that HSC-derived leukocytes in vaginal tract may be dominant for production of CCL2 before 24 h pi, which is then replaced by CCL2 produced from resident cells of vaginal tract. In contrast, CCL3 as well as CCL4 and CCL5 showed secretion patterns that gradually increased from 24 h pi with maximum secretion at 48 h pi in KO-WT chimeras, suggesting that secretion of CCL3, together with several CCR5-binding CC chemokines, may be more dependent on resident cells after 24 h pi and subsequently involved in inducing infiltration of NK and T cells in the vaginal tract. However, CXCL1, a ligand of CXCR2 for neutrophil infiltration, intriguingly showed highest levels in KO-KO chimeras, which displayed increased infiltration of CD11b^+^Ly-6G^hi^ neutrophils. Ultimately, these results indicate that early infiltration of CD11b^+^Ly-6C^hi^ monocytes depends on CCL2 produced from HSC-derived leukocytes before 24 h pi, whereas CCL3, CCL4, and CCL5 produced from resident cells after 48 h pi are dominant regulators that induce infiltration of NK cells and CD11c^+^ DCs, and then T cells at a later phase.

### Orchestrated response of HSC-derived leukocytes and resident cells for consecutive recruitment of Ly-6C^hi^ monocytes and NK cells via CC chemokines

**Fig 6:**
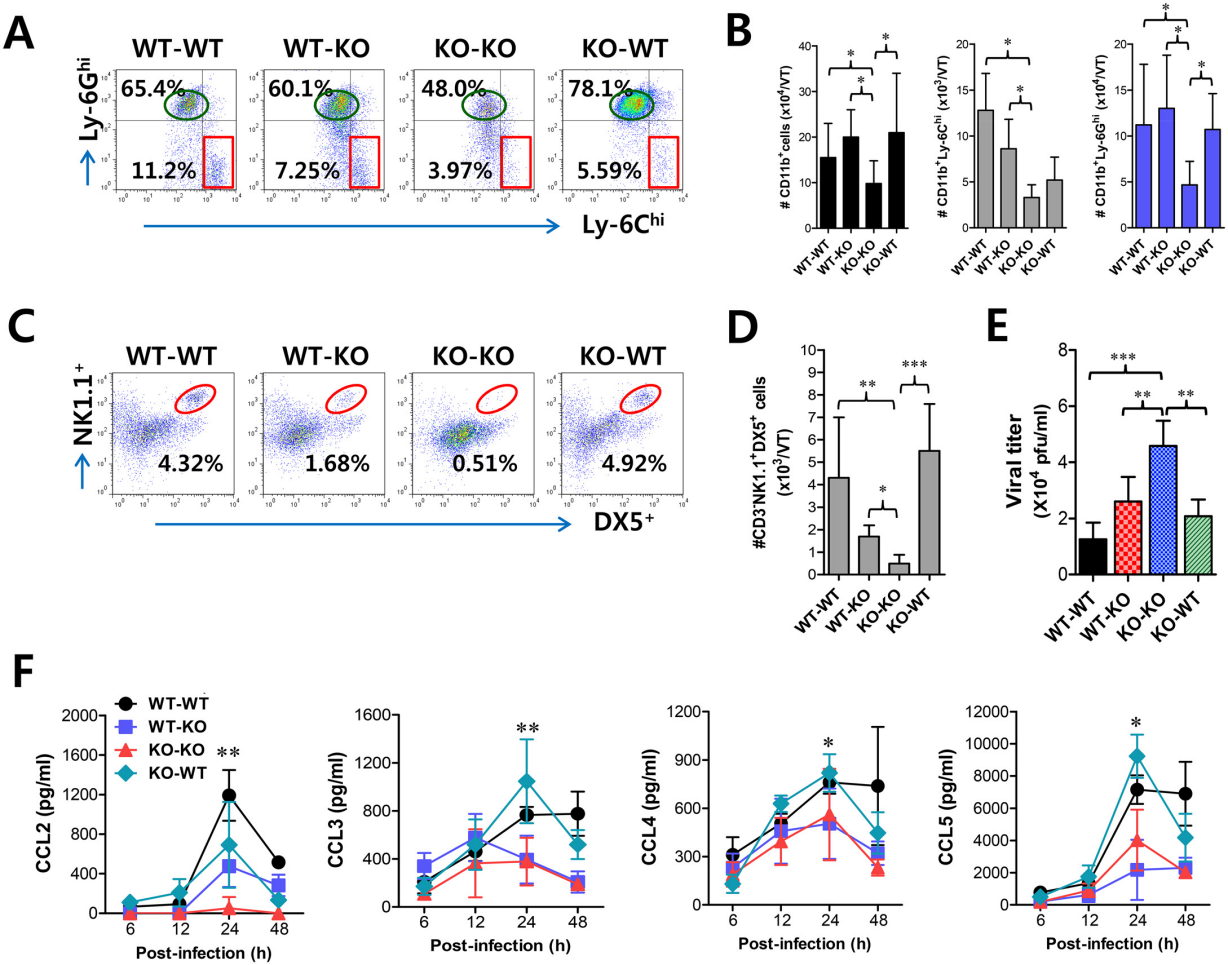
CCL2 produced from infiltrated leukocytes derived from HSC lineage plays a dominant role in mucosal recruitment of CD11b^+^Ly-6C^hi^ monocytes. BM cells from BL/6 (WT) or *CCL2* KO (KO) mice were grafted to lethally irradiated BL/6 or *CCL2* KO recipient mice, which were infected i.vag. with HSV-1. **(A,B)** CD11b^+^Ly-6C^hi^ infiltration of infected recipients in vaginal tract. Cells were prepared from the vaginal tract by collagenase digestion at 24 h pi and subcellular proportions of CD11b^+^Ly-6C^hi^ and CD11b^+^Ly-6G^hi^ leukocytes were determined using flow cytometric analysis. **(C,D)** Frequency and absolute number of infiltrated NK cells in vaginal tract of the recipients. Cells prepared from the vaginal tract of the recipients were used to analyze the frequency (C) and total number (D) of CD3^−^NK1.1^+^DX5^+^ NK cells at 48 h pi. **(E)** Viral titers in vaginal lavages. Viral titers in vaginal lavages collected at 48 h pi were determined by plaque assay. **(F)** Chemokine secretion in vaginal lavages of the recipients. The levels of chemokine proteins were determined by CBA using vaginal lavages at 6, 12, 24, and 48 h pi. Values in dot-plots represent the average percentages of each population derived from four independent samples, and data in graphs represent the average ± SD of values derived from three individual experiments (*n* = 4–5). *, *p*<0.05; **, *p*<0.01; ***, *p*<0.001 compared with the level of the indicated group.

To further confirm the differential dependence of CD11b^+^Ly-6C^hi^ monocyte and NK cell infiltration on CC chemokines produced by HSC-derived leukocytes and mucosal resident cells, we used BM chimeric model of WT BL/6 and CCL2 KO mice. Since CCL2 KO recipients of WT BM donor cells (WT-KO) displayed a significantly higher frequency of vaginal CD11b^+^Ly-6C^hi^ monocytes compared to CCL2 KO recipients of CCL2 KO BM donor cells (KO-KO) and WT recipients of CCL2 KO BM donor cells (KO-WT), CCL2 produced by HSC-derived leukocytes appeared to play a dominant role in the early recruitment of CD11b^+^Ly-6C^hi^ monocytes in vaginal tract (Fig 6A). However, CC chemokines produced by vaginal resident cells were also considered to have a residual role in the recruitment of CD11b^+^Ly-6C^hi^ monocytes because WT-KO chimeras still showed a lower frequency of vaginal CD11b^+^Ly-6C^hi^ monocytes than WT recipients of WT BM donor cells (WT-WT). Supporting these findings, when the absolute number of CD11b^+^Ly-6C^hi^ monocytes in the vaginal tract was determined, a significant role of CCL2 produced by HSC-derived leukocytes was evident in the early recruitment of CD11b^+^Ly-6C^hi^ monocytes in the vaginal tract (Fig 6B). Moreover, CCL2 produced from HSC-derived leukocytes and resident cells was shown to partially contribute to vaginal recruitment of CD11b^+^Ly-6G^hi^ neutrophils, because KO-KO chimeras showed a decreased number of vaginal CD11b^+^Ly-6G^hi^ neutrophils compared to WT-KO and KO-WT chimeric models (Fig 6B). In addition, our data revealed that CCL2 played a certain role in recruiting NK cells, because KO-KO chimeras showed diminished NK cell frequency and number compared to WT-WT chimeras (Fig 6C and 6D). However, recruitment of CD3^−^NK1.1^+^DX5^+^ NK cells in vaginal tract appeared to dominantly depend on CC chemokines produced by resident cells together with CCL2, because KO-WT chimeras displayed a comparable frequency of NK cells to WT-WT chimeras. Also, WT-KO chimeras showed a higher frequency of NK cells in the vaginal tract than KO-KO chimeras, which indicates that CCL2 produced by HSC-derived leukocytes contributes in part to NK cell recruitment. Viral burden in vaginal lavages reflected the differential dependence of CD11b^+^Ly-6C^hi^ monocytes and NK cells on CCL2 produced from HSC-derived and resident cells (Fig 6E). We also examined the levels of secreted CC and CXC chemokines in vaginal lavages of four CCL2 KO BM chimeric models. As expected, WT-WT chimeras showed the highest CCL2 levels, which peaked at 24 h pi and declined by 48 h pi, whereas a considerable amount of secreted CCL2 protein was detected in WT-KO chimeras with a delayed pattern that started at around 24 h pi and gradually increased until 48 h pi (Fig 6F), indicating that early infiltrated HSC-derived leukocytes producing CCL2 may be evident from around 24 h pi. In contrast with *IFNAR* KO BM chimera experiment, secretion of CCL2 protein was evident at higher levels in KO-WT chimeras at 24 h pi compared to WT-KO chimeras. This implies that vaginal resident cells could compensate for the CCL2 supply at a certain point in the absence of CCL2 production by infiltrated leukocytes. Other CC chemokines (CCL3, CCL4, CCL5) that are involved in NK and Th CD4^+^ T cell recruitment were detected at higher levels in WT-WT and KO-WT chimeras compared to WT-KO and KO-KO chimeras, suggesting that the supply of those CC chemokines is mostly accomplished by vaginal resident cells after 24 h pi. Moreover, maximum secretion of CCR5 ligands (CCL3, CCL4, CCL5) was observed in vaginal tract of KO-WT chimera at around 24 h pi, which implies that partial CCL2 ablation could change sequential production of other chemokines, due to compensation roles. Collectively, these results indicate that CCL2 produced by HSC-derived leukocytes is predominantly responsible for the accumulation of CD11b^+^Ly-6C^hi^ monocytes within 24 pi, whereas recruitment of NK cells in the vaginal tract appears to be mediated by the CC chemokine pool achieved by vaginal resident cells from 24 to 48 h pi, together with CCL2.

### Critical role of IFN-I signaling on tissue resident CD11b^hi^F4/80^hi^ macrophages and CD11c^hi^EpCAM^+^ DCs in the initial production of CCL2 proteins

**Fig 7:**
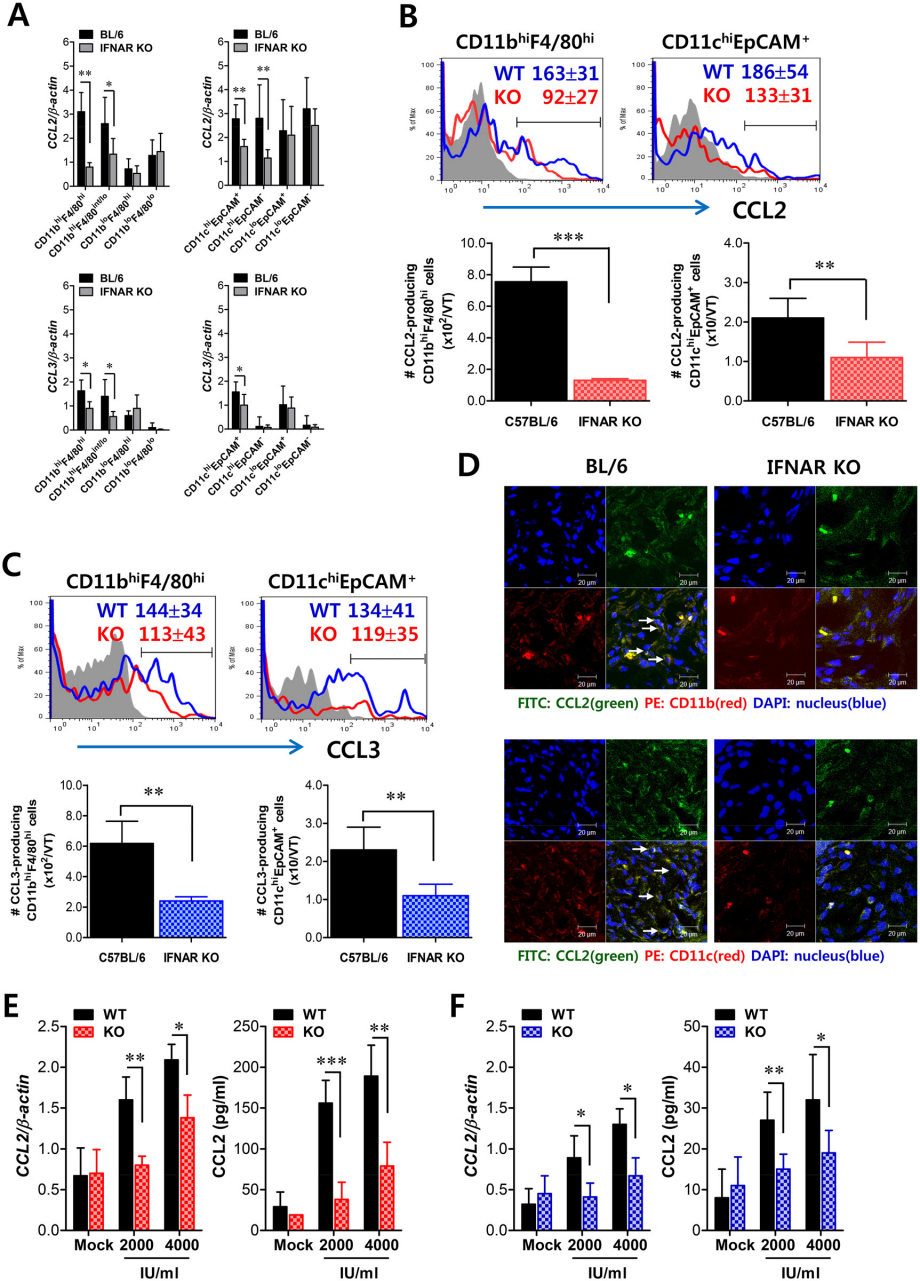
Critical role of IFN-I signaling on resident CD11b^hi^F4/80^hi^ macrophages and CD11c^hi^EpCAM^+^ DCs in the production of initial CCL2 protein for early migration of CD11b^+^Ly-6C^hi^ monocytes. **(A)** Early expression of CCL2 and CCL3 by sorted CD11b^hi^F4/80^hi^ macrophages and CD11c^hi^EpCAM^+^ DCs. Subsets of macrophages (CD11b^hi^F4/80^hi^, CD11b^hi^F4/80^int/lo^, CD11b^lo^F4/80^hi^, and CD11b^lo^F4/80^lo^) and DCs (CD11c^hi^EpCAM^+^, CD11c^hi^EpCAM^−^, CD11c^lo^EpCAM^+^, and CD11c^lo^EpCAM^−^) were sorted 12 h after mucosal HSV-1 infection, and CCL2 and CCL3 expression was analyzed with real-time qRT-PCR. **(B,C)** Regulation of CCL2 and CCL3 production from CD11b^hi^F4/80^hi^ macrophages and CD11c^hi^EpCAM^+^ DCs by IFN-I signaling. CD11b^hi^F4/80^hi^ macrophages and CD11c^hi^EpCAM^+^ DCs producing CCL2 (B) and CCL3 (C) were determined by flow cytometric analysis at 12 h pi. Blue line, HSV-1-infected BL/6; Red line, HSV-1-infected IFNAR KO; Gray line, mock-infected. **(D)** Confocal microscopic analysis of early CCL2 production by resident CD11b^+^ macrophages and CD11c^+^ DCs. CD11b^+^ macrophages and CD11c^+^ DCs producing CCL2 protein were visualized by confocal microscopy at 12 h pi. CD11b^+^ macrophages and CD11c^+^ DCs producing CCL2 protein are denoted by white arrows. **(E,F)** Essential role of IFN-I signaling in the production of CCL2 protein from CD11b^hi^F4/80^hi^ macrophages and CD11c^hi^EpCAM^+^ DCs. Vaginal CD11b^hi^F4/80^hi^ macrophages (E) and CD11c^hi^EpCAM^+^ DCs (F) isolated from WT and *IFNAR* KO mice were stimulated with recombinant IFN-α (2,000 and 4,000 IU/ml) for 6 h. The expression and production of CCL2 were determined by real-time qRT-PCR and CBA using stimulated cells and collected culture media, respectively. Data represent the average ± SD of values derived from three individual experiments (*n* = 4–5). *, *p*<0.05; **, *p*<0.01; ***, *p*<0.001 compared with the level of the indicated group.

Since early infiltrated HSC-derived leukocytes are considered the main cellular regulators in the recruitment of CD11b^+^Ly-6C^hi^ monocytes via CCL2, unsolved questions still remain: What induces the initial infiltration of these leukocytes producing CCL2? What are the initial regulators of leukocyte recruitment, including CD11b^+^Ly-6C^hi^ monocytes? Because infiltrated CD11b^+^Ly-6C^hi^ monocytes are also the main producers of CCL2 protein, we assumed that the seeded amount of CCL2 produced by certain resident cells in vaginal tract might induce initial recruitment of CD11b^+^Ly-6C^hi^ monocytes, which subsequently provide amplified recruitment of more CD11b^+^Ly-6C^hi^ monocytes via CCL2. To this end, we examined CCL2 and CCL3 expression in tissue resident CD11b^hi^ and CD11c^hi^ cell subpopulations at 12 h pi, before the recruitment of CD11b^+^Ly-6C^hi^ monocytes peaked. Interestingly, initial expression of CCL2 was dominantly achieved in CD11b^hi^F4/80^hi^ macrophages and CD11c^hi^EpCAM^+^ DCs sorted from vaginal tract of WT BL/6 mice, compared to those of *IFNAR* KO mice (Fig 7A). Also, other CD11b^hi^ myeloid cells (CD11b^hi^F4/80^lo^) and CD11c^hi^ DCs (CD11c^hi^EpCAM^−^), but not CD11b^lo^ and CD11c^lo^ cells, showed significantly higher levels of CCL2 expression in WT BL/6 compared to *IFNAR* KO mice. In addition, CCL3 expression was also apparent in CD11b^hi^F4/80^hi^ macrophages and CD11c^hi^EpCAM^+^ DCs derived from WT BL/6 mice, even though levels of CCL3 expression were much lower than those of CCL2. To confirm these findings, we enumerated CCL2-producing CD11b^hi^F4/80^hi^ macrophages and CD11c^hi^EpCAM^+^ DCs in vaginal tract of both WT BL/6 and IFNAR KO mice at 12 h pi. As expected, increased expression of CCL2 protein in vaginal CD11b^hi^F4/80^hi^ macrophages and CD11c^hi^EpCAM^+^ DCs of WT BL/6 mice was observed, and a higher number of CCL2-producing CD11b^hi^F4/80^hi^ macrophages and CD11c^hi^EpCAM^+^ DCs was detected in vaginal tract of WT BL/6 mice, compared with *IFNAR* KO mice (Fig 7B). Similarly, vaginal CD11b^hi^F4/80^hi^ macrophages and CD11c^hi^EpCAM^+^ DCs derived from WT BL/6 mice showed higher expression of CCL3 compared to those of *IFNAR* KO mice (Fig 7C). The production of CCL2 protein by vaginal CD11b^hi^F4/80^hi^ macrophages and CD11c^hi^EpCAM^+^ DCs was further visualized using confocal microscopy (Fig 7D). In consistent, CCL2-producing CD11b^+^ and CD11c^+^ cells were detected at a higher frequency in vaginal tract of WT BL/6 mice than *IFNAR* KO mice. Also, F4/80^+^ cells producing CCL2 protein were detected at a higher frequency in the vaginal tract of WT BL/6 than *IFNAR* KO mice (S4 Fig). Since IFN-I signaling plays an important role in inducing CCL2 protein by stimulation with IFN-I proteins produced from epithelial cells through IFI-16 and STING recognition upon HSV-1 infection [34,35], we examined the expression of CCL2 from vaginal CD11b^hi^F4/80^hi^ macrophages and CD11c^hi^EpCAM^+^ DCs sorted from WT BL/6 and *IFNAR* KO mice upon stimulation with IFN-α protein. As expected, CD11b^hi^F4/80^hi^ macrophages and CD11c^hi^EpCAM^+^ DCs derived from vaginal tract of WT BL/6 mice showed higher expression of CCL2 protein than those of *IFNAR* KO mice (Fig 7E and 7F), even though CCL2 expression in CD11c^hi^EpCAM^+^ DCs was detected at lower levels than CD11b^hi^F4/80^hi^ macrophages. Similarly, other CC chemokines (CCL3, CCL4, CCL5), but not CXC chemokines, were expressed with slightly higher levels in CD11b^hi^F4/80^hi^ macrophages and CD11c^hi^EpCAM^+^ DCs derived from vaginal tract of WT BL/6 mice, compared to those of *IFNAR* KO (S5 Fig). Collectively, these results suggest that tissue resident CD11b^hi^F4/80^hi^ macrophages and CD11c^hi^EpCAM^+^ DCs are candidates for supplying the initial CCL2 protein upon stimulation by IFN-I produced from infected epithelial cells, thereby inducing early infiltration of CD11b^+^Ly-6C^hi^ monocytes into mucosal tissues.

### Tissue resident CD11b^hi^F4/80^hi^ macrophages and CD11c^hi^EpCAM^+^ DCs play a role in establishing early orchestrated infiltration of Ly-6C^hi^ monocytes and NK cells

**Fig 8:**
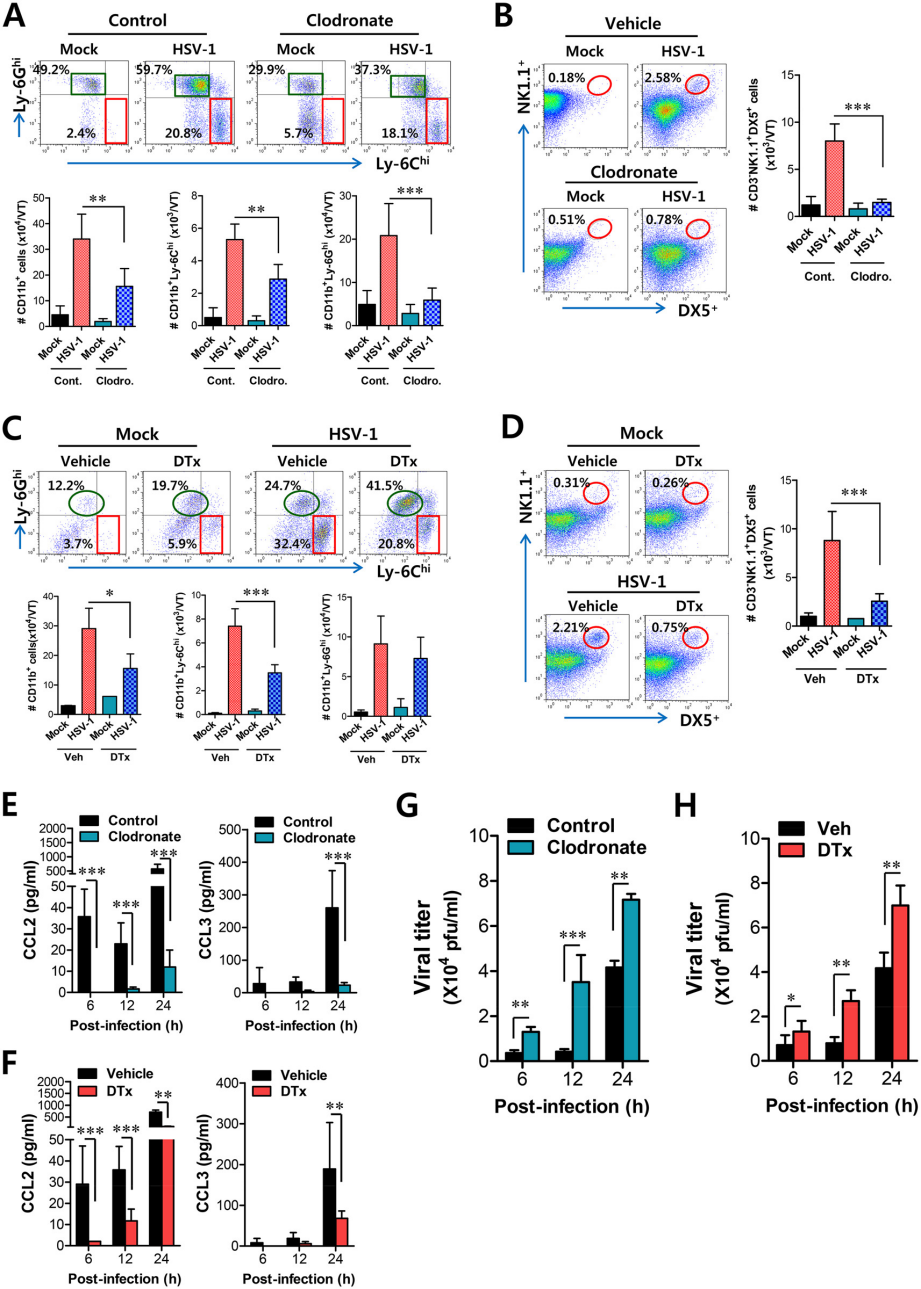
Resident CD11b^hi^F4/80^hi^ macrophages and CD11c^hi^ DCs play a key role in initiating migration-based self-amplification of CD11b^+^Ly-6C^hi^ monocytes through very early production of CCL2 protein. **(A)** Crucial role of resident CD11b^hi^F4/80^hi^ macrophages in inducing early recruitment of CD11b^+^Ly-6C^hi^ monocytes. BL/6 mice were treated with clodronate liposomes to deplete CD11b^+^ macrophages and infected i.vag. with HSV-1. The infiltration of CD11b^+^Ly-6C^hi^ monocytes was determined by flow cytometric analysis at 24 h pi. **(B)** Regulation of NK cell infiltration by CD11b^+^F4/80^+^ macrophages. CD3^−^NK1.1^+^DX5^+^ NK cells in vaginal tract were analyzed in clodronate-treated mice at 48 h pi. **(C)** Regulation of initial CD11b^+^Ly-6C^hi^ monocyte infiltration by CD11c^hi^ DCs. CD11c-DTR Tg mice were treated with DT to deplete CD11c^hi^ DCs and infected i.vag. with HSV-1. The infiltration of CD11b^+^Ly-6C^hi^ monocytes was determined by flow cytometric analysis at 24 h pi. **(D)** Regulation of NK cell infiltration by CD11c^hi^ DCs. CD3^−^NK1.1^+^DX5^+^ NK cells in the vaginal tract were analyzed in DT-treated CD11c-DTR mice at 48 h pi. **(E,F)** Resident CD11b^hi^F4/80^hi^ macrophages and CD11c^hi^ DCs provide very early CCL2 production. The levels of CCL2 and CCL3 proteins in vaginal lavages were determined in clodronate- and DT-treated mice at 6, 12, and 24 h pi. **(G,H)** Viral replication in vaginal tract of mice depleted for CD11b^hi^F4/80^hi^ macrophages and CD11c^hi^ DCs. Viral titers in vaginal tract of clodronate- and DT-treated mice were determined by plaque assay at 6, 12, and 24 h pi. Data in the bar chart represent the average ± SD of values derived from three individual experiments (*n* = 4–5). *, *p*<0.05; **, *p*<0.01; ***, *p*<0.001 compared with the level of the indicated group.

Since tissue resident CD11b^hi^F4/80^hi^ macrophages and CD11c^hi^EpCAM^+^ DCs were proposed to be the initial key cell populations for recruiting early CD11b^+^Ly-6C^hi^ monocytes for migration-based self-amplification, we investigated the contribution of these cell populations in establishing early orchestrated infiltration of CD11b^+^Ly-6C^hi^ monocytes and NK cells in vaginal tract. To examine the role of CD11b^hi^F4/80^hi^ macrophages we used the depletion model of CD11b^hi^F4/80^hi^ cells using clodronate liposome, as WT BL/6 mice showed an approximately 60% reduction in CD11b^hi^F4/80^hi^ macrophages after administration of clodronate liposomes (S6A and S6B Fig). Ablation of tissue resident CD11b^hi^F4/80^hi^ macrophages reduced the accumulated number of infiltrated CD11b^+^Ly-6C^hi^ monocytes, but the frequency of CD11b^+^Ly-6C^hi^ monocytes was not obviously changed after gating on CD11b^+^ cells because of the effect of clodronate liposomes on total CD11b^+^ myeloid cells (Fig 8A). Thus, the accumulated number of total CD11b^+^ and CD11b^+^Ly-6G^hi^ granulocytes was also reduced. Since clodronate liposomes affect total CD11b^+^ myeloid cells, including tissue resident CD11b^hi^F4/80^hi^ macrophages, we also used GdCl_3_, which has been known as macrophage-selective inhibitor [36]. Our data revealed that GdCl_3_ administration selectively reduced the frequency and accumulated number of CD11b^+^Ly-6C^hi^ monocytes in vaginal tract (S7A Fig). Also, the recruitment of NK cells in vaginal tract was apparently decreased in both frequency and accumulated number by the inhibition of tissue resident CD11b^hi^F4/80^hi^ macrophages using cloronate liposomes (Fig 8B) and GdCl_3_ (S7B Fig). In addition, we used CD11c-DTR mice that allow conditional DC depletion upon DT injection (S6C and S6D Fig) to examine the role of CD11c^hi^EpCAM^+^ DCs in the recruitment of early CD11b^+^Ly-6C^hi^ monocytes. Consistently, ablation of CD11c^hi^EpCAM^+^ DCs reduced the frequency and accumulated number of CD11b^+^Ly-6C^hi^ monocytes and NK cells in vaginal tract (Fig 8C and 8D). These results suggest that tissue resident CD11b^hi^F4/80^hi^ macrophages and CD11c^hi^EpCAM^+^ DCs play an important role in recruiting the early infiltration of CD11b^+^Ly-6C^hi^ monocytes to foster the orchestrated infiltration of subsequent leukocytes.

To further understand the role of CD11b^hi^F4/80^hi^ macrophages and CD11c^hi^EpCAM^+^ DCs in recruiting initial CD11b^+^Ly-6C^hi^ monocytes for migration-related self-amplification, we examined CCL2 and CCL3 production in vaginal tracts of mice in which CD11b^hi^F4/80^hi^ macrophages and CD11c^hi^EpCAM^+^ DCs were depleted. Ablation of tissue resident CD11b^hi^F4/80^hi^ macrophages and CD11c^hi^EpCAM^+^ DCs diminished production of CCL2 protein as early as 6 h pi, but CCL3 production was reduced at 24 h pi (Fig 8E and 8F) with a delayed pattern, which appeared to result from decreased infiltration of CD11b^+^Ly-6C^hi^ monocytes. In support of these findings, decreased infiltration of early CD11b^+^Ly-6C^hi^ monocytes in vaginal tract of mice depleted for CD11b^hi^F4/80^hi^ macrophages and CD11c^hi^EpCAM^+^ DCs was closely associated with enhanced replication of HSV-1 (Fig 8G and 8H). Selective inhibition of CD11b^hi^F4/80^hi^ macrophages by GdCl3 also resulted in an enhanced viral burden in vaginal lavages (S7C Fig). Therefore, these results indicate that tissue resident CD11b^hi^F4/80^hi^ macrophages and CD11c^hi^EpCAM^+^ DCs in vaginal tract are key cellular regulators for the initial recruitment of CD11b^+^Ly-6C^hi^ monocytes, thereby establishing an orchestrated environment of subsequent leukocyte infiltration through their migration-based self-amplification to provide early viral clearance.

## Discussion

Although extensive studies have focused on the antiviral nature of IFN-I, its role in the generation of innate immune cells derived from HSC lineages was only recently described. In particular, IFN-I signaling appears to be critical for the recruitment and activation of innate immune cells, including CD11b^+^Ly-6C^hi^ monocytes and NK cells, in inflammatory tissues [29,30]. However, the critical regulators of IFN-I–dependent recruitment of CD11b^+^Ly-6C^hi^ monocytes and NK cells and their cellular source were not defined. Our data show that IFN-I signals govern the recruitment and activation of CD11b^+^Ly-6C^hi^ and NK cells, which is associated with susceptibility to HSV-1 mucosal infection. An intriguing finding is that the sequential recruitment of CD11^+^Ly-6C^hi^ monocytes and then NK cells into mucosal tissue appears to depend on the CCL2-CCL3 cascade mediated by HSC-derived leukocytes and resident cells, respectively. Furthermore, tissue resident CD11b^hi^F4/80^hi^ macrophages and CD11c^hi^EpCAM^+^ DCs were involved in producing the initial CCL2 for migration-based self-amplification of rapidly infiltrated CD11b^+^Ly-6C^hi^ monocytes through stimulation by IFN-I produced from infected epithelial cells. To the best of our knowledge, this is the first report that deciphers the cellular and molecular cascade pathway to regulate IFN-I-dependent recruitment of CD11b^+^Ly-6C^hi^ monocytes and NK cells into inflammatory mucosal tissues.

Orchestrated mobilization of innate and adaptive immune cells between the vaginal epithelium and the draining (iliac) LNs during mucosal HSV-1 infection is critical for the control of viral replication, thereby preventing CNS invasion. However, the redundancy of chemokine recognition by immune cells sometimes makes the interpretation of results difficult. The chemokine receptor CCR2 is expressed in CD11b^+^Ly-6C^hi^ monocytes as well as a subset of B, NK, NKT cells, pDCs, and activated T cells [37–39]. However, since the number of B, NKT cells, pDCs, and activated T cells in vagina mucosa of *CCR2* KO mice is comparable to that of WT mice following mucosal HSV infection [28], CCR2 is thought to play a role in the recruitment of CD11b^+^Ly-6C^hi^ monocytes and NK cells. In addition, NK cell recruitment is also regulated by other CC chemokines, including CCL3, CCL4, and CCL5, as a result of expression of CCR5 on NK cells [40–42], and CXCL10, a ligand of CXCR3, was suggested to mediate NK cell mobilization in vaginal tissue as a principal chemoattractant molecule that is liberated early after genital HSV-2 infection [43]. Consequently, CCR2 is considered the main chemokine receptor involved in the recruitment of CD11b^+^Ly-6C^hi^ monocytes, whereas several chemokine receptors appear to be involved in the recruitment of NK cells. Likewise, our data demonstrate that the production of CCL2, a ligand of CCR2, is kinetically associated with the vaginal recruitment of CD11b^+^Ly-6C^hi^ monocytes, which peaked at 24 h pi. CD11b^+^Ly-6C^hi^ monocytes egress massively from bone marrow into the bloodstream in a CCR2-dependent manner and are recruited via the CCL2-CCR2 axis into infected sites, where they exert direct antimicrobial activities or further differentiate into inflammatory DCs [24,25]. However, the cellular source of CCL2 for the recruitment of CD11b^+^Ly-6C^hi^ monocytes into mucosal tissues was not clearly defined. Our experiments using *IFNAR* KO BM chimeric models demonstrate that some leukocytes derived from the HSC lineage play a dominant role in the recruitment of CD11b^+^Ly-6C^hi^ monocytes into vaginal tissues as well as iliac LNs because only *IFNAR* KO mice reconstituted by WT BM cells (WT-KO) showed comparable recruitment of CD11b^+^Ly-6C^hi^ monocytes into vaginal and iliac LN tissues to that of WT recipients of WT BM cells (WT-WT). This result contrasts starkly with the fact that IFN-I signals on corneal resident cells play an important role in recruiting CD11b^+^Ly-6C^hi^ monocytes in the corneal area [13], but is in line with a report that IFN-I signaling on the hematopoietic cell lineage plays a dominant role in leukocyte recruitment into inflammatory lung tissues after influenza infection [29]. Supportively, CCL2 production in vaginal tissues of WT-KO BM chimeric model gradually increased up to 24 h pi, after which CCL2 level decreased. This implies that recruitment of CD11b^+^Ly-6C^hi^ monocytes may be governed by other chemokines produced from resident cells after 24 h pi or that CCL2 pool produced up to 24 h is sufficient to recruit major CD11b^+^Ly-6C^hi^ monocytes. The former speculation is supported by the fact that CCL2 level in vaginal lavages of *CCL2* KO-WT BM chimera gradually increased after 24 h pi, suggesting that resident cells such as epithelial cells resistant to γ-irradiation might produce CCL2 and other CC chemokines with delayed patterns compared to HSC-derived leukocytes. However, as the recruitment of CD11b^+^Ly-6C^hi^ monocytes peaked at 24 h pi, CCL2 produced from HSC-derived leukocytes within 24 h pi is considered to be responsible for the major infiltration of CD11b^+^Ly-6C^hi^ monocytes. Our experiments using *CCL2* KO BM chimeras in part support this, because *CCL2* KO recipients of WT BM cells (WT-KO) elicited a higher frequency of CD11b^+^Ly-6C^hi^ monocytes than KO-KO and KO-WT BM chimeras, indicating that CCL2 produced from HSC-derived leukocytes plays a dominant role in recruiting early CD11b^+^Ly-6C^hi^ monocytes in vaginal tissues. Consequently, the turning point at which the recruitment of CD11b^+^Ly-6C^hi^ monocytes is replaced by recruitment of other cell populations, including NK cells and CD11c^+^ DCs, appeared to occur between 24 and 48 h pi.

In addition to CD11b^+^Ly-6C^hi^ monocytes, neutrophils are a major population of innate immune cells that are involved in immunopathology [44,45], although their role in viral infection remains controversial. According to our data, it is likely that IFN-I signaling is a negative regulator in the recruitment of CD11b^+^Ly-6G^+^ neutrophils into vaginal tissues. Indeed, whereas IFN-I signaling seems to play a general role in promoting CD11b^+^Ly-6C^hi^ monocytes [29–31], its effect on CD11b^+^Ly-6G^+^ neutrophil trafficking and function are quite divergent between different infection models [46,47]. We show here that *IFNAR* KO mice produced higher amounts of CXCL1 and CXCL2, which are the main ligands of CXCR2 expressed on neutrophils. It was recently reported that IFN-I abrogates the recruitment of neutrophils to ganglia by directly suppressing CXCL2 expression by monocytes [48]. This is in line with our finding that infiltrated CD11b^+^Ly-6C^lo^ cells sorted from *IFNAR* KO mice, but not CD11b^+^Ly-6C^hi^ monocytes, expressed high levels of CXCL1 and CXCL2 compared to those from WT mice. However, the most interesting result is that the recruitment of CD11b^+^Ly-6G^+^ neutrophils depends mostly on resident cells, because only WT mice reconstituted by *IFNAR* KO BM cells showed identical levels of infiltrated neutrophils to the KO-KO chimeras. This indicates that IFN-I signaling on HSC-derived leukocytes suppresses the recruitment of neutrophils in vaginal tissues following HSV-1 infection, thereby ameliorating aberrant immunopathology. Considering that *IFNAR* KO mice lack neutrophil recruitment in inflamed tissues in bacterial infection models [49], it seems likely that IFN-I signaling in HSC-derived leukocytes performs divergent roles to recruit specific innate cells depending on the context and specific details of the infection model.

In contrast to the recruitment of CD11b^+^Ly-6C^hi^ monocytes, and somewhat intriguingly, NK cell recruitment into vaginal and iliac LN tissues is delayed and dependent on resident cells such as epithelial and stromal cells that are resistant to γ-irradiation. It is possible that CCL3, CCL4, and CCL5, which are ligands of CCR5 expressed on NK cells, are the main regulators of NK cell recruitment because their expression was kinetically associated with the recruitment of NK cells. Also, our data support the residual role of HSC-derived leukocytes in recruiting NK cells in vaginal and iliac LN tissues because of the increased accumulation of NK cells in *IFNAR* KO recipients of WT BM cells (WT-KO) compared with KO-KO BM chimera. The role of CCL3 and CCL5 in NK cell recruitment has been extensively described [40–42], and our data are further strengthened by striking evidence that decreased CCL3 production in *IFNAR* KO mice results in reduced NK cell accumulation in a model of murine cytomegalovirus (MCMV) infection [41]. Considering that the cellular source of CC chemokines that recruit NK cells in vaginal tissue following HSV infection is not yet defined, our data provide new insight into the dominant role of CC chemokines produced from resident cells such as infected and uninfected epithelial cells that are stimulated by IFN-I produced from infected neighbor cells [50]. Nevertheless, CCL3 and CCL5 produced from infiltrated CD11b^+^Ly-6C^hi^ and CD11b^+^Ly-6C^lo^ monocytes probably do not play a residual role in recruiting vaginal NK cells because there was no difference in CCL3 and CCL5 production between monocytes sorted from WT and *IFNAR* KO mice. Instead, CCL2 produced from CD11b^+^Ly-6C^hi^ monocytes appears to share responsibility in recruiting NK cells with CCL3 and CCL5 produced from resident cells, because *CCL2* KO recipients of WT BM cells (WT-KO) showed a higher number of vaginal NK cells than KO-KO BM chimeras. Despite debatable issue, CCL2-CCR2 axis is believed to play a certain role in recruiting NK cells to infected sites [51], which is consistent with our result that *CCL2* KO recipients of *CCL2* KO BM cells showed reduced infiltration of NK cells in vaginal tissues following HSV-1 infection. Consequently, the recruitment of latecomer NK cells in vaginal tissue appears to be partly regulated by the earlier recruitment of first-mover CD11b^+^Ly-6C^hi^ monocytes, which mostly depends on HSC-derived leukocytes.

**Fig 9:**
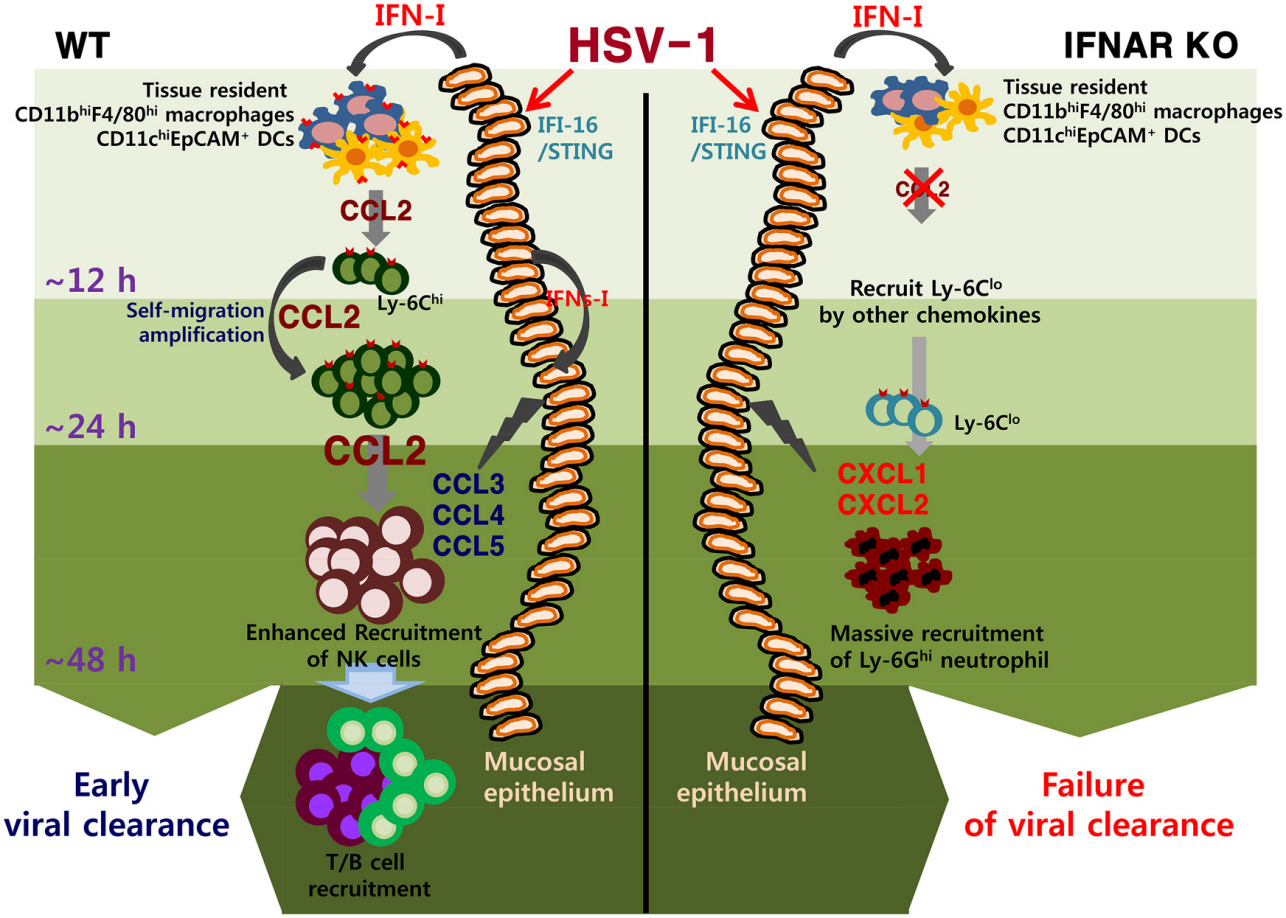
IFN-I–dependent cascade pathway for concerted recruitment of CD11b^+^Ly-6C^hi^ monocytes and NK cells in mucosal tissues following HSV-1 infection. At the very initial phase of infection, CCL2 is initially produced from tissue resident CD11b^hi^F4/80^hi^ macrophages and CD11c^hi^EpCAM^+^ DCs through stimulation with IFN-I proteins produced from mucosal epithelium via the action of IFI-16 and STING in response to vaginal infection with HSV-1, thereby recruiting very early CD11b^+^Ly-6C^hi^ monocytes within 12 h pi. Subsequently, the early infiltrated CD11b^+^Ly-6C^hi^ monocytes contribute to migration-based self-amplification through the supply of higher levels of CCL2 at around 24 h pi. Delayed production of other CC chemokines (CCL3, CCL4, CCL5) from infected and uninfected epithelial cells, together with CCL2 provided by monocytes, then contributes to the recruitment of late-comer NK cells at 48 h pi. The lack of an initial CCL2 supply in *IFNAR* KO mice results in reduced recruitment of CD11b^+^Ly-6C^hi^ monocytes; instead, CXC chemokines (CXCL1, CXCL2) primarily produced from resident cells and CD11b^+^Ly-6C^lo^ monocytes probably induce massive recruitment of CD11b^+^Ly-6G^+^ neutrophils.

CCL2 chemokine is widely studied because of its upregulation in autoimmune disease, tumors, and infections. Although CCL2 production has been shown to be regulated by TNF-α and STAT2 signals [52,53], CCL2 production can be induced by stimulation with IFN-I alone through an IFN-responsive element in the *CCL2* upstream promoter [13]. Following HSV-1 mucosal infection, initial viral replication is limited to mucosa epithelium of the vaginal tract, where IFN-I is produced through the action of IFI-16/p204 and the downstream adaptor molecule STING [13,34,35,50]. This IFN-I appears to stimulate neighboring epithelial cells and tissue resident immune cells to induce the expression of CCL2 [13,50]. As another interesting finding, our data suggest the importance of vaginal tissue resident CD11b^hi^F4/80^hi^ macrophages and CD11c^hi^EpCAM^+^ DCs in providing the initial CCL2 protein responsible for recruiting early CD11b^+^Ly-6C^hi^ monocytes within 12 h pi, although CD11c^−^EpCAM^+^ epithelial cells can also produce CCL2 protein through IFN-I stimulation [13,50]. It is likely that the amount of CCL2 protein produced from vaginal tissue resident CD11b^hi^F4/80^hi^ macrophages and CD11c^hi^EpCAM^+^ DCs is low, due to basally low number of these cell populations in vaginal tissue. However, the initial CCL2 may be able to recruit early CD11b^+^Ly-6C^hi^ monocytes, which in turn provide more CCL2 protein to establish self-amplification of CD11b^+^Ly-6C^hi^ monocyte migration. This notion is strengthened by a previous study showing that CD11b^+^F4/80^+^ macrophages in the liver provide initial CCL2 protein to recruit NK cells in a MCMV infection model [51]. The vaginal tract is composed of three principal layers: (i) the mucosa epithelium composed of stratified nonkeratinized squamous epithelial tissue, (ii) the submucosa of vascularized connective tissue, and (iii) the muscularis layer composed of smooth muscle [54]. CD11b^hi^F4/80^hi^ macrophages and CD11c^hi^EpCAM^+^ DCs, which are responsive to stimulation by IFN-I [54], are strategically localized in both the stratified squamous epithelial layer and the submucosal lamina propria. Therefore, the data presented here suggest that IFN-I produced from initially infected epithelial cells stimulate tissue resident CD11b^hi^F4/80^hi^ macrophages and CD11c^hi^EpCAM^+^ DCs to produce initial CCL2 protein, thereby recruiting early CD11b^+^Ly-6C^hi^ monocytes from the bloodstream and bone marrow within 12 h pi. Subsequently, early infiltrated CD11b^+^Ly-6C^hi^ monocytes may establish migration-based self-amplification through the supply of a high amount of CCL2 protein, which peaks at around 24 h pi. Subsequently, levels of CC chemokines (CCL3, CCL4, and CCL5) produced from resident cells such as infected and uninfected epithelial cells gradually increase and peak at 48 h pi; these chemokines are responsible for the recruitment of NK cells, together with CCL2 produced by CD11b^+^Ly-6C^hi^ monocytes (Fig 9). However, a lack of initial CCL2 supply from tissue resident CD11b^hi^F4/80^hi^ macrophages and CD11c^hi^EpCAM^+^ DCs in *IFNAR* KO mice results in diminished recruitment of CD11b^+^Ly-6C^hi^ monocytes and subsequent NK cells. Instead, CXC chemokines produced from IFNAR-deficient resident cells and CD11b^+^Ly-6C^lo^ monocytes is likely to induce massive recruitment of CD11b^+^Ly-6G^hi^ neutrophils into vaginal tissues.

The role of CD11b^+^Ly-6C^hi^ monocytes in controlling parasitic and bacterial infections is well described [6,24–28], but the contribution of these cells in HSV infection has only recently begun to grab the attention [13,28]. The majority of studies suggest a predominant role of neutrophils and NK cells in containing HSV replication during the innate immune response [55,56], whereas the mechanism of the antimicrobial activity by CD11b^+^Ly-6C^hi^ monocytes is ambiguous. Although our data discount direct antiviral action of IFN-I on HSV-1 replication [57,58], we surmise a coordinated role of early infiltrated CD11b^+^Ly-6C^hi^ monocytes in establishing orchestrated mobilization of innate immune cells to contain HSV-1 replication in vaginal tissues. Furthermore, considering that IFN-I signaling plays an important role in HSC proliferation and differentiation [59–61], *IFNRA* KO mice may contain different cell constitution from WT mice during infection. Specific cell populations directly affected by IFN-I during hematopoiesis and their contributions to unique cell population in peripheral tissues has been recently described [29]. Supportively, IFN-I–dependent differentiation of CD11b^+^Ly-6C^hi^ monocytes producing CCL2 appears to play a critical role in establishing the orchestrated mobilization of innate cells, whereas the differentiation of CD11b^+^Ly-6C^lo^ monocytes producing CXCL1, IL-23, IL-10, and TGF-β is likely to induce aberrant sequential inflammation in vaginal tissue of *IFNAR* KO mice. This finding is also strengthened by the recent report that IFN-I attenuates neutrophil infiltration to prevent severe tissue damage caused by uncontrolled inflammation [48]. In conclusion, our findings elucidate a detailed IFN-I-dependent pathway that establishes the orchestrated mobilization of CD11b^+^Ly-6C^hi^ monocytes and NK cells through consecutive production of CCL2 and CCL3 chemokines from HSC-derived leukocytes and epithelium-resident cells. Our data further imply that the cascade responses of resident-to-hematopoietic-to-resident cells for cytokine-to-chemokine-to-cytokine production to recruit innate immune cells are critical for the attenuation of HSV replication in inflamed tissues.

## Materials and Methods

### Ethics statement

All animal experiments described in the present study were conducted at Chonbuk National University according to the guidelines set by the Institutional Animal Care and Use Committees (IACUC) of Chonbuk National University and were pre-approved by the Ethical Committee for Animal Experiments of Chonbuk National University (Permission code 2013–0028). Animal research protocol in this study followed the guideline set up by the nationally recognized Korea Association for Laboratory Animal Sciences (KALAS).

### Animals

Female C57BL/6 (H-2^b^) mice (6–8 weeks old) were purchased from Samtako (O-San, Korea). *CCL2* knockout (KO) and *CD11c-DTR* transgenic (Tg) mice (B6.FVB-Tg Itgax-DTR/EGFP 57Lan/J [DTR]) were obtained from The Jackson Laboratories (Bar Harbor, ME, USA). *IFNAR* KO mice deficient in the receptor for type I IFNs (IFN-α/β) were described elsewhere [29]. All mice were genotyped and bred in the animal facilities of Chonbuk National University.

### Cells, viruses, and in vivo viral infection

The HSV-1 McKrae strain was propagated in Vero cells (CCL81; ATCC, Manassas, VA, USA) using DMEM supplemented with 2.5% FBS, penicillin (100 U/ml), and streptomycin (100 U/ml). The Vero cells were infected with HSV-1 at a multiplicity of infection (MOI) of 0.01 and incubated in a CO_2_ incubator for 1 h at 37°C with shaking at 15-min intervals. After absorption, the inoculum was removed, and 5 ml of maintenance medium containing 2.5% FBS was added. Approximately 2–3 dpi, cultures of the host cells showing an 80–90% cytophatic effect were harvested and titrated by conventional plaque assay using Vero cells. The virus stocks were stored in aliquots at -80°C until use. For in vivo viral challenge, the mice were pretreated with progesterone to synchronize their estrous cycles as described earlier [62,63]. Briefly, mice were subcutaneously (s.c.) injected with medroxyprogesteron 17-acetate (Depoprovera [DP]; Sigma-Aldrich, St. Louis, MO, USA) at 2 mg per mouse. Five days after injection of DP, the mice were challenged intravaginally (i.vag.) with the HSV-1 McKrae strain (1×10^6^ PFU). Infected mice were examined daily for vaginal inflammation, neurological illness, and death, as described previously [63]. Mice were scored 1 to 5 depending on the clinical severity of disease (0, no change; 1, mild inflammation; 2, moderate swelling; 3, severe inflammation; 4, paralysis; 5, death).

### Antibodies and reagents

The monoclonal antibodies used for flow cytometric analysis and other experiments were purchased from eBioscience (San Diego, CA, USA), BD Bioscience (San Diego, CA, USA), and R&D Systems (Minneapolis, MN, USA), which included: FITC-conjugated anti-mouse CD3ε (145-2C11), CD40 (HM40-3), CD80 (16-10A1), CD86 (GL1), MHC-II (M5/114.15.2), CD11c (N418), F4/80 (BM8), and CCL2 (2H5); PE-conjugated anti-mouse IFN-γ (XMG1.2), granzyme B (NGZB), CD11b (M1/70), CD326 (EpCAM) (G8.8), CCR2 (475301), and CXCR2 (242216); PE-Cy-7–conjugated anti-mouse NK1.1 (PK136), PerCP-Cy5.5–conjugated anti-mouse Ly-6C (HK1.4), PerCP-conjugated anti-mouse CCL3(MIP-1 alpha) (DNT3CC); allophycocyanin (APC)-conjugated anti-mouse Ly-6G (1A8) and F4/80 (BM8); biotin-conjugated anti-mouse pan-NK cells, CD49b-Integrin alpha 2 (DX5), F4/80 (BM8); and streptavidin-conjugated APC.

### Quantitation of viral burden and cytokine/chemokine expression

#### Quantitative real-time PCR for viral burden and cytokine/chemokine expression

Viral burden and cytokine/chemokine expression were assessed by real-time qPCR using genomic DNA extracted from collected tissues and real-time qRT-PCR using total RNA extracted from collected tissues and sorted cells, respectively. After extraction of genomic DNA and total RNA, real-time qPCR was performed using a CFX96 Real-Time PCR Detection system (Bio-Rad Laboratories, Hercules, CA, USA). To determine the expression of cytokine and chemokine mRNAs, total RNA was subjected to reverse transcription with High-Capacity cDNA Reverse Transcription Kits (Applied Biosystems, Foster City, CA, USA) for real-time qPCR. The reaction mixture contained 2 μl of template cDNA, 10 μl of 2× SYBR Primix Ex Taq, and 200 nM specific primers (S1 Table) at a final volume of 20 μl. The reaction mixes were denatured at 95°C for 30 s and then subjected to 45 cycles of 95°C for 5 s and 60°C for 20 s. After the reaction cycle was completed the temperature was increased from 65°C to 95°C at a rate of 0.2°C/15 s and fluorescence was measured every 5 s to construct a melting curve. A control sample that contained no template DNA was run with each assay, and all determinations were performed at least in duplicate to ensure reproducibility. The authenticity of the amplified product was determined by melting curve analysis. Viral burden was expressed as viral DNA copy number per microgram of genomic DNA, and the expression of cytokines and chemokines was expressed as relative fold expression after normalization to the housekeeping gene β-actin. All data were analyzed using Bio-Rad CFX Manager, version 2.1 analysis software (Bio-Rad Laboratories).

#### Cytokine bead array (CBA)

The levels of chemokine proteins in vaginal lavages and sera were measured by the cytometric bead array (CBA) specific for CCL2 (MCP-1), CCL3 (MIP-1α), CCL4 (MIP-1β), CCL5 (RANTES), CCL7 (MCP-3), and CXCL1 (KC), according to the manufacturer’s protocols (BD Bioscience).

### Analysis of infiltrated leukocytes in vaginal tract and iliac lymph node

Vaginal tract and iliac LN were collected from WT and *IFNAR* KO mice infected with HSV-1 McKrae strain (1×10^6^ PFU/mouse) at 12, 24, 48, and 72 h pi. Leukocytes that had infiltrated into the vaginal tract and iliac LN were harvested, homogenized by gently pressing through a 100-mesh tissue sieve, and digested with 25 μg/ml collagenase type IV (Worthington Biochem, Freefold, NJ, USA) in RPMI medium for 1 h at 37°C in a shaking incubator. Cells were counted and stained for CD11b, Ly6G, Ly6C, CD3, CD4, CD8, and NK1.1 with directly conjugated antibodies (eBioscience) for 30 min at 4°C. Finally, the cells were fixed with 10% formaldehyde. Data collection and analysis were performed with a FACSCalibur flow cytometer (Becton Dickson Medical Systems, Sharon, MA, USA) and FlowJo (Tree Star, San Carlos, CA, USA) software.

### Analysis of NK cell activity

The activity of NK cells was assessed by their capacity to produce IFN-γ and granzyme B (GrB) following brief stimulation with phorbol 12-myristate 13-acetate (PMA) and ionomycin (Sigma-Aldrich). Vaginal leukocytes were prepared from WT and *IFNAR* KO mice at 48 h pi and stimulated with PMA and ionomycin in the presence of monensin (2 μM) to induce the expression of IFN-γ (PMA 50 ng/ml plus ionomycin 750 ng/ml for 2 h) and GrB (PMA 50 ng/ml plus ionomycin 750 ng/ml for 4 h). After stimulation, cells were surface-stained using FITC anti-mouse-CD3ε, PE-Cy7 anti-mouse NK1.1, biotin-conjugated anti-mouse pan-NK cells and anti-CD49b (DX5) antibodies, and streptavidin-APC for 30 min at 4°C. The cells were washed twice with FACS buffer containing monensin. After fixation, the cells were permeabilized with 1× permeabilization buffer (eBioscience) and stained intracellularly with PE-conjugated anti-mouse IFN-γ (XMG1.2) and granzyme B (16G6) antibodies in permeabilization buffer for 30 min at 4°C. Finally, the cells were washed twice with PBS and analysis was performed with a FACS Calibur flow cytometer (Becton Dickson Medical Systems) using FlowJo (Tree Star) software.

### Generation of bone marrow chimeric mice

C57BL/6, *IFNAR* KO, and *CCL2* KO mice were γ-irradiated with one dose of 950 rads. Within 12 h, recipient mice were reconstituted with bone marrow (BM) cells (1.5×10^7^ cells/mouse) derived from C57BL/6, *IFNAR* KO, and *CCL2* KO donor mice, respectively. The recipient mice were given sulfamethoxazole and trimethoprim in their drinking water for the next 10 days. Recipient mice were allowed to reconstitute their hematopoietic stem cell (HSC) population for 4 to 6 weeks. Chimerism was confirmed prior to experimental use. BM chimeric mice were infected i.vag. with HSV-1 McKrae strain (1×10^6^ or 1×10^7^ PFU/mouse) and sacrificed at the indicated days pi. Cells that infiltrated into vaginal tissues and iliac LN were analyzed by flow cytometry.

### Confocal microscopy

For fluorescence staining, mice were infected i.vag. with HSV-1 McKrae strain (1×10^6^ PFU/mouse) and sacrificed at 12 h pi. Vaginal tracts were collected and frozen in optimum cutting temperature (OCT) compound. Sections of 6–7 μm thickness were cut, air-dried, and fixed in cold solution (1:1 mixture of acetone and methanol) for 15 min at -20°C. After washing with PBS three times, non-specific binding was blocked with 10% normal goat serum and cells were permeabilized with 0.1% Triton X-100. Staining was performed by incubating sections overnight in moist chambers at 4°C with biotin-conjugated anti-mouse myeloid cell marker CD11b, DC marker CD11c, or macrophage marker F4/80 plus FITC-conjugated anti-mouse CCL2. Primary antibodies were detected with PE-conjugated streptavidin. Nuclei were counterstained with DAPI (4'6-diamidino-2-phenylindole; Sigma-Aldrich). Fluorescence was observed using a confocal laser scanning microscope (Carl Zeiss, Zena, Germany).

### Depletion of localized CD11b^hi^F4/80^hi^ macrophages and CD11c^hi^EpCAM^+^ DCs

Temporal depletion of localized CD11b^hi^F4/80^hi^ macrophages in the vaginal tract was achieved by both i.v. and i.vag. administration of clodronate liposome or PBS liposome (250 μl for i.v. and 10 μl/VT for i.vag.) 24 h before challenge. For depletion of CD11c^hi^EpCAM^+^ DCs, CD11c-DTR Tg mice were administered diphtheria toxin (DT) via both i.v. and i.vag. routes at 12 and 24 h before challenge (9 ng/g for i.v. and 320 ng/VT for i.vag.). In some experiments, gadolinium chloride (III) (GdCl_3_; 10 mg/kg for i.v. and 10 mg/VT for i.vag. at -24 h pi) was administered as a selective inhibitor of macrophages [36]. Macrophage and DC depletion in vaginal tract was confirmed by flow cytometry. Mice with depleted macrophages and DCs in vaginal tract were infected with HSV-1 McKrae strain (1×10^7^ PFU/mouse).

### Statistical analysis

All data were expressed as the average ± standard deviation, and statistically significant differences between groups were analyzed by unpaired two-tailed Student’s *t*-test for ex vivo experiments and immune cell analysis or ANOVA and post-hoc test for multiple comparisons of the mean. The statistical significance of viral burden and in vivo cytokine gene expression was evaluated by Mann-Whitney test or unpaired two-tailed Student’s t-test. Kaplan-Meier survival curves were analyzed by the log-rank test. A *p* value ≤0.05 was considered significant. All data were analyzed using Prism software (GraphPadPrism4, San Diego, CA, USA).

## Supporting Information

S1 Fig IFN-I signaling is essential to control CNS invasion of HSV-1. BL/6 (*n* = 8) or *IFNAR* KO (*n* = 9) were challenged i.vag. with HSV-1 (1×10^6^ PFU/mouse). (A,B) Survival rate and clinical score. Surviving mice were examined daily up to 21 dpi (A), and clinical severity was recorded at the indicated dpi (B) according to scoring guidelines. (C) Infectious viral shedding in primary target tissues. Infectious virus titer was measured by plaque assay using vaginal lavages collected at the indicated dpi. (D-H) Viral burden in target and lymphoid tissues. Viral burden in vaginal tract (D), iliac LN (E), spleen (F), spinal cord (G), and brain (H) of BL/6 or *IFNAR* KO mice infected i.vag. with HSV-1 was assessed by real-time PCR at the indicated dpi. The viral burden was expressed as viral DNA copy number per microgram of genomic DNA. Each symbol represents the levels of an individual mouse; the horizontal line indicates the median of each group.
(PDF)

S2 Fig IFN-I signaling is essential to establish early orchestrated expression of cytokines and chemokines in primary inflammation tissues. Heatmap showing the expression of cytokines and chemokines in each tissue. The expression levels of cytokines and chemokines were assessed by real-time qRT-PCR using total RNA extracted from vaginal tract (VT), iliac LN (ILN), spleen (Spl), spinal cord (SC), and brain of infected BL/6 or *IFNAR* KO mice at the indicated dpi. The expression of each cytokine and chemokine is normalized to that of -actin and is displayed as the average of three individual experiments (*n* = 4–5), according to the indicated color on a log_2_ scale.
(PDF)

S3 Fig Effect of IFN-I signaling on the differentiation of NK cells in various tissues. Uninfected BL/6 or *IFNAR* KO mice were sacrificed and leukocytes were prepared for the analysis of CD3^−^NK1.1^+^DX5^+^ NK cells in various tissues including blood, liver, ILN (iliac LN), and vaginal tract (VT). (A) NK cell frequency. (B) Total number of CD3^−^NK1.1^+^DX5^+^ NK cells in each tissue. Values in dot-plots represent the average percentages of CD3^−^NK1.1^+^DX5^+^ NK cells after gating on CD3-negative cells. Data in bar charts represent the average ± SD derived from three individual experiments (*n* = 5). **, *p*<0.01 compared with the levels of IFNAR KO mice.
(PDF)

S4 Fig Confocal microscopic analysis of early CCL2 production by resident F4/80^+^ macrophages. Resident F4/80^+^ macrophages producing CCL2 protein were visualized by confocal microscopy at 12 h pi. F4/80^+^ macrophages producing CCL2 protein are denoted by white arrows.
(PDF)

S5 Fig The role of IFN-I signaling in the production of CC and CXC chemokines from CD11b^hi^F4/80^hi^ macrophages and CD11c^hi^EpCAM^+^ DCs. Vaginal CD11b^hi^F4/80^hi^ macrophages (A) and CD11c^hi^EpCAM^+^ DCs (B) isolated from WT and *IFNAR* KO mice were stimulated with recombinant IFN-α (2,000 and 4,000 IU/ml) for 6 h. The expression of CC and CXC chemokines were determined by real-time qRT-PCR. Data represent the average ± SD derived from three individual experiments (*n* = 4–5).
(PDF)

S6 Fig Depletion of CD11b^hi^F4/80^hi^ macrophages and CD11c^hi^ DCs. (A and B) BL/6 mice were treated with clodronate liposomes via both intravenous (i.v.) and i.vag. routes. Resident CD11b^hi^F4/80^hi^ macrophages in vaginal tract were determined by flow cytometric analysis 24 h after clodronate treatment. (C and D) CD11c-DTR Tg mice were administered DT via both intraperitoneal (i.p.) and i.vag. routes. Resident CD11c^hi^ DCs were determined by flow cytometric analysis 24 h after DT treatment. Values in the representative dot-plots denote the average percentages of vaginal macrophages and DCs derived from at least four independent samples, and data in the bar chart represent the average ± SD derived from three individual experiments (*n* = 4–5). **, *p*<0.01; ***, *p*<0.001 compared with the levels of the indicated group.
(PDF)

S7 Fig Selective inhibition of resident CD11b^hi^F4/80^hi^ macrophages by GdCl_3_ reduces early infiltration of CD11b^+^Ly-6C^hi^ monocytes and NK cells. BL/6 mice were treated with GdCl_3_ via both i.v. and i.vag. routes, and were infected i.vag. with HSV-1 24 h after GdCl_3_ treatment. (A) Selective reduction of CD11b^+^Ly-6C^hi^ monocyte infiltration by GdCl_3_-mediated inhibition of resident CD11b^hi^F4/80^hi^ macrophages. Infiltrated CD11b^+^Ly-6C^hi^ monocytes were determined by flow cytometric analysis at 24 h pi. (B) Reduced recruitment of NK cells in GdCl_3_-treated mice. CD3^−^NK1.1^+^DX5^+^ NK cells in vaginal tract were analyzed in GdCl_3_-treated mice at 48 h pi. (C) Viral replication in vaginal tract of GdCl_3_-treated mice. Viral titers in vaginal lavages of GdCl_3_-treated mice were determined by plaque assay at 6, 12, and 24 h pi. Values in the representative dot-plots denote the average percentages of Ly-6C^hi^ monocytes, Ly-6G^hi^ granulocytes and NK cells derived from at least four independent samples, and data in the bar chart represent the average ± SD derived from three individual experiments (*n* = 4–5). **, *p*<0.01; ***, *p*<0.001 compared with the level of the indicated group.
(PDF)

S1 Table Specific primers for PCR amplification of cytokines and chemokines. (PDF)
