# Supplementary material for: Distinct Upstream Role of Type I IFN Signaling in Hematopoietic Stem Cell-Derived and Epithelial Resident Cells for Concerted Recruitment of Ly-6Chi Monocytes and NK Cells via CCL2-CCL3 Cascade
Source: PLoS Pathog. 2015 Nov 30;11(11):e1005256. doi: 10.1371/journal.ppat.1005256 (PMC4664252; doi:10.1371/journal.ppat.1005256)
Supplement: S1 Fig — BL/6 (n = 8) or IFNAR KO (n = 9) were challenged i.vag. with HSV-1 (1×10^6^ PFU/mouse). (A,B) Survival rate and clinical score. Surviving mice were examined daily up to 21 dpi (A), and clinical severity was recorded at the indicated dpi (B) according to scoring guidelines. (C) Infectious viral shedding in primary target tissues. Infectious virus titer was measured by plaque assay using vaginal lavages collected at the indicated dpi. (D-H) Viral burden in target and lymphoid tissues. Viral burden in vaginal tract (D), iliac LN (E), spleen (F), spinal cord (G), and brain (H) of BL/6 or IFNAR KO mice infected i.vag. with HSV-1 was assessed by real-time PCR at the indicated dpi. The viral burden was expressed as viral DNA copy number per microgram of genomic DNA. Each symbol represents the levels of an individual mouse; the horizontal line indicates the median of each group. (PDF) [file ppat.1005256.s001.pdf]

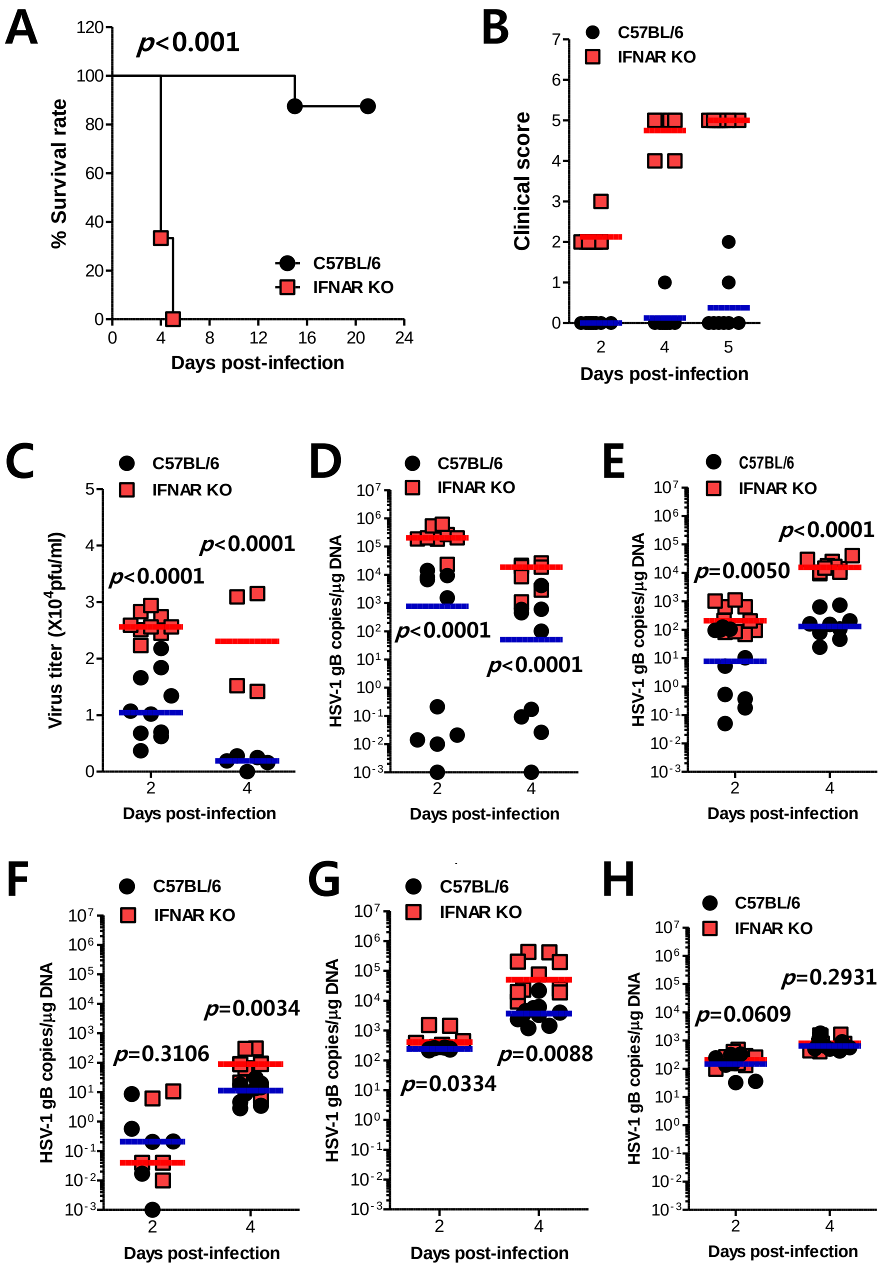

S1 Fig. IFN-I signaling is essential to control CNS invasion of HSV-1. BL/6 (n=8) or *IFNAR* KO (n=9) were challenged i.vag. with HSV-1 (1×10^6 PFU/mouse). (A,B) Survival rate and clinical score. Surviving mice were examined daily up to 21 dpi (A), and clinical severity was recorded at the indicated dpi (B) according to scoring guidelines. (C) Infectious viral shedding in primary target tissues. Infectious virus titer was measured by plaque assay using vaginal lavages collected at the indicated dpi. (D-H) Viral burden in target and lymphoid tissues. Viral burden in vaginal tract (D), iliac LN (E), spleen (F), spinal cord (G), and brain (H) of BL/6 or *IFNAR* KO mice infected i.vag. with HSV-1 was assessed by real-time PCR at the indicated dpi. The viral burden was expressed as viral DNA copy number per microgram of genomic DNA. Each symbol represents the levels of an individual mouse; the horizontal line indicates the median of each group.
