# Supplementary material for: Distinct Upstream Role of Type I IFN Signaling in Hematopoietic Stem Cell-Derived and Epithelial Resident Cells for Concerted Recruitment of Ly-6Chi Monocytes and NK Cells via CCL2-CCL3 Cascade
Source: PLoS Pathog. 2015 Nov 30;11(11):e1005256. doi: 10.1371/journal.ppat.1005256 (PMC4664252; doi:10.1371/journal.ppat.1005256)
Supplement: S2 Fig — Heatmap showing the expression of cytokines and chemokines in each tissue. The expression levels of cytokines and chemokines were assessed by real-time qRT-PCR using total RNA extracted from vaginal tract (VT), iliac LN (ILN), spleen (Spl), spinal cord (SC), and brain of infected BL/6 or IFNAR KO mice at the indicated dpi. The expression of each cytokine and chemokine is normalized to that of -actin and is displayed as the average of three individual experiments (n = 4–5), according to the indicated color on a log2 scale. (PDF) [file ppat.1005256.s002.pdf]

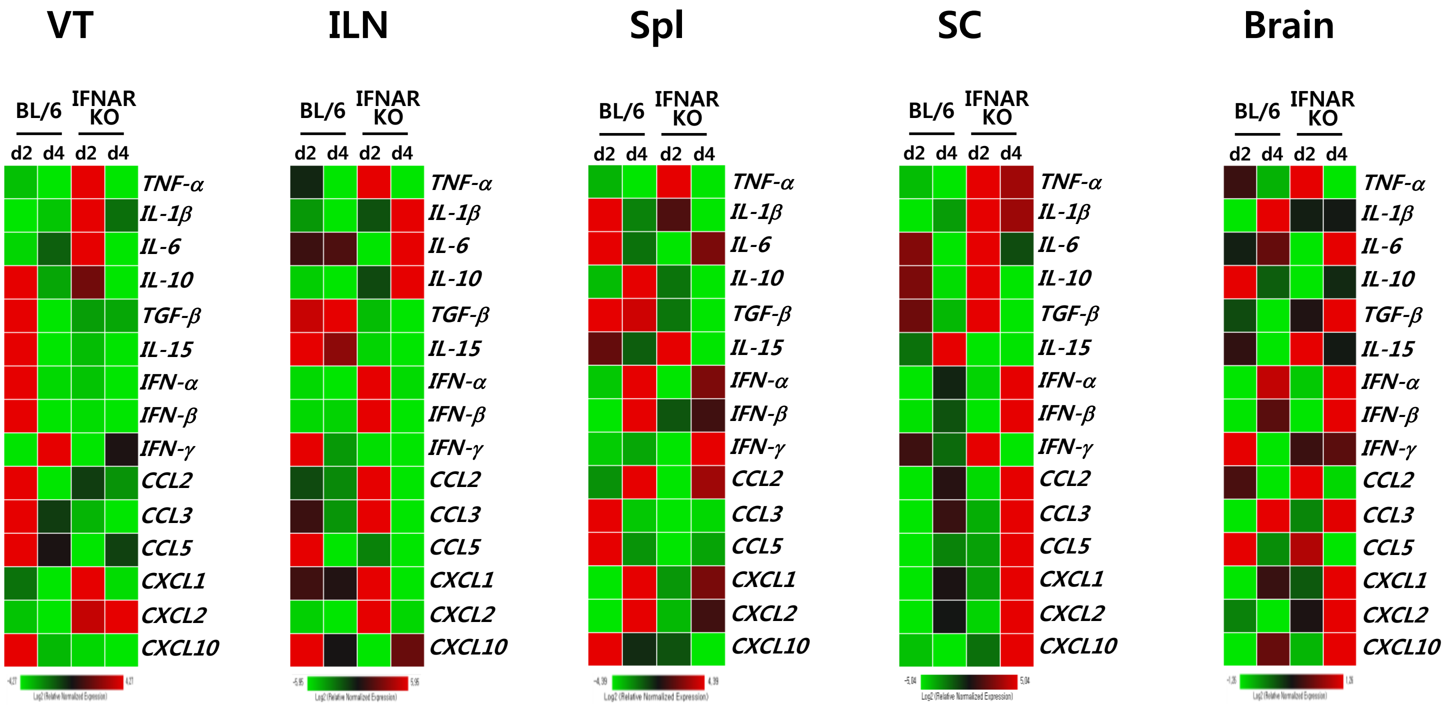

S2 Fig. IFN-I signaling is essential to establish early orchestrated expression of cytokines and chemokines in primary inflammation tissues. Heatmap showing the expression of cytokines and chemokines in each tissue. The expression levels of cytokines and chemokines were assessed by real-time qRT-PCR using total RNA extracted from vaginal tract (VT), iliac LN (ILN), spleen (Spl), spinal cord (SC), and brain of infected BL/6 or *IFNAR* KO mice at the indicated dpi. The expression of each cytokine and chemokine is normalized to that of β-actin and is displayed as the average of three individual experiments (*n*=4-5), according to the indicated color on a $\log_2$ scale.
