# Supplementary material for: Distinct Upstream Role of Type I IFN Signaling in Hematopoietic Stem Cell-Derived and Epithelial Resident Cells for Concerted Recruitment of Ly-6Chi Monocytes and NK Cells via CCL2-CCL3 Cascade
Source: PLoS Pathog. 2015 Nov 30;11(11):e1005256. doi: 10.1371/journal.ppat.1005256 (PMC4664252; doi:10.1371/journal.ppat.1005256)
Supplement: S3 Fig — Uninfected BL/6 or IFNAR KO mice were sacrificed and leukocytes were prepared for the analysis of CD3−NK1.1+DX5+ NK cells in various tissues including blood, liver, ILN (iliac LN), and vaginal tract (VT). (A) NK cell frequency. (B) Total number of CD3−NK1.1+DX5+ NK cells in each tissue. Values in dot-plots represent the average percentages of CD3−NK1.1+DX5+ NK cells after gating on CD3-negative cells. Data in bar charts represent the average ± SD derived from three individual experiments (n = 5). **, p<0.01 compared with the levels of IFNAR KO mice. (PDF) [file ppat.1005256.s003.pdf]

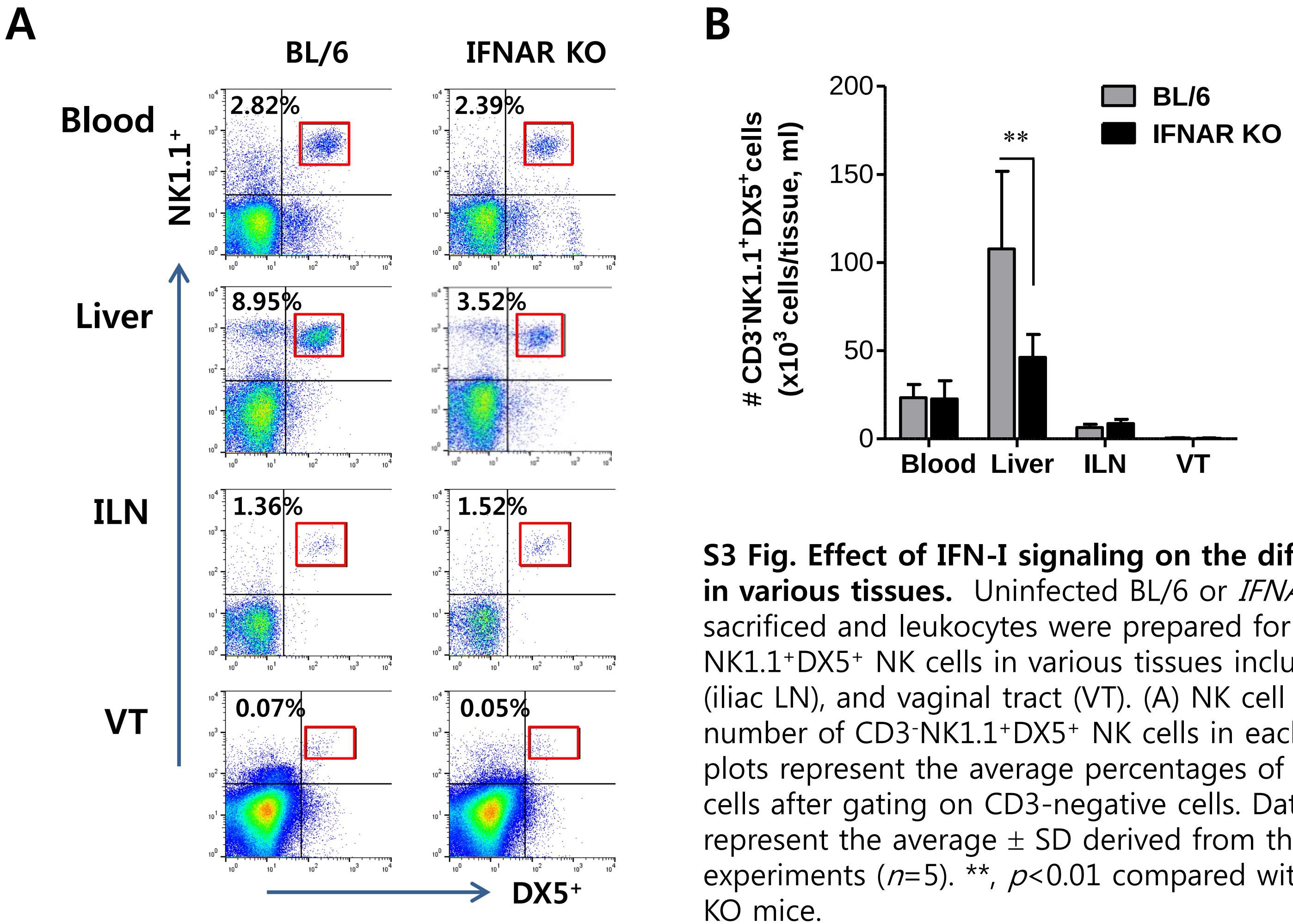

S3 Fig. Effect of IFN-I signaling on the differentiation of NK cells in various tissues. Uninfected BL/6 or *IFNAR* KO mice were sacrificed and leukocytes were prepared for the analysis of $CD3^-$ $NK1.1^+DX5^+$ NK cells in various tissues including blood, liver, ILN (iliac LN), and vaginal tract (VT). (A) NK cell frequency. (B) Total number of $CD3^-NK1.1^+DX5^+$ NK cells in each tissue. Values in dot-plots represent the average percentages of $CD3^-NK1.1^+DX5^+$ NK cells after gating on CD3-negative cells. Data in bar charts represent the average ± SD derived from three individual experiments (n=5). **, p<0.01 compared with the levels of IFNAR KO mice.
