# Supplementary material for: Distinct Upstream Role of Type I IFN Signaling in Hematopoietic Stem Cell-Derived and Epithelial Resident Cells for Concerted Recruitment of Ly-6Chi Monocytes and NK Cells via CCL2-CCL3 Cascade
Source: PLoS Pathog. 2015 Nov 30;11(11):e1005256. doi: 10.1371/journal.ppat.1005256 (PMC4664252; doi:10.1371/journal.ppat.1005256)
Supplement: S4 Fig — Resident F4/80+ macrophages producing CCL2 protein were visualized by confocal microscopy at 12 h pi. F4/80+ macrophages producing CCL2 protein are denoted by white arrows. (PDF) [file ppat.1005256.s004.pdf]

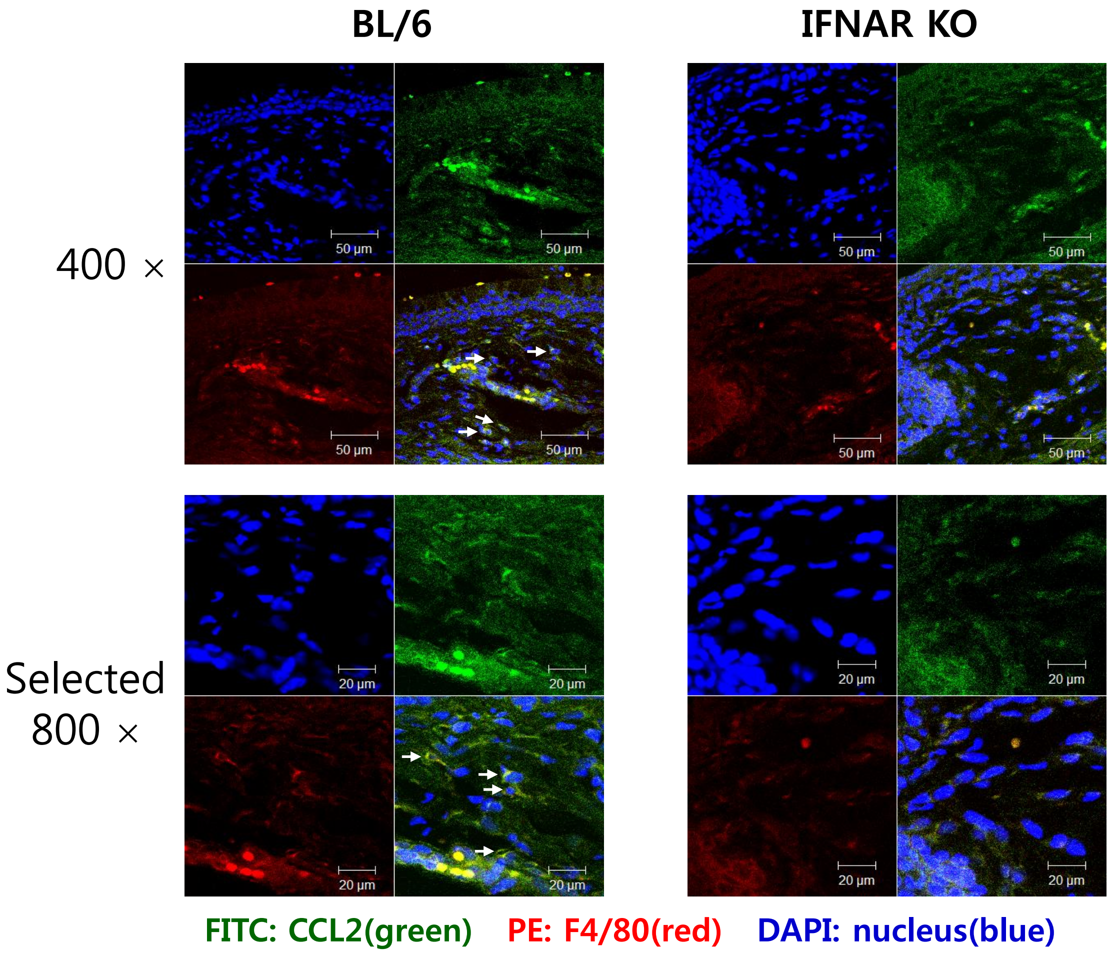

S4 Fig. Confocal microscopic analysis of early CCL2 production by resident F4/80$^+$ macrophages. Resident F4/80$^+$ macrophages producing CCL2 protein were visualized by confocal microscopy at 12 h pi. F4/80$^+$ macrophages producing CCL2 protein are denoted by white arrows.
