# Supplementary material for: Distinct Upstream Role of Type I IFN Signaling in Hematopoietic Stem Cell-Derived and Epithelial Resident Cells for Concerted Recruitment of Ly-6Chi Monocytes and NK Cells via CCL2-CCL3 Cascade
Source: PLoS Pathog. 2015 Nov 30;11(11):e1005256. doi: 10.1371/journal.ppat.1005256 (PMC4664252; doi:10.1371/journal.ppat.1005256)
Supplement: S5 Fig — Vaginal CD11bhiF4/80hi macrophages (A) and CD11chiEpCAM+ DCs (B) isolated from WT and IFNAR KO mice were stimulated with recombinant IFN-α (2,000 and 4,000 IU/ml) for 6 h. The expression of CC and CXC chemokines were determined by real-time qRT-PCR. Data represent the average ± SD derived from three individual experiments (n = 4–5). (PDF) [file ppat.1005256.s005.pdf]

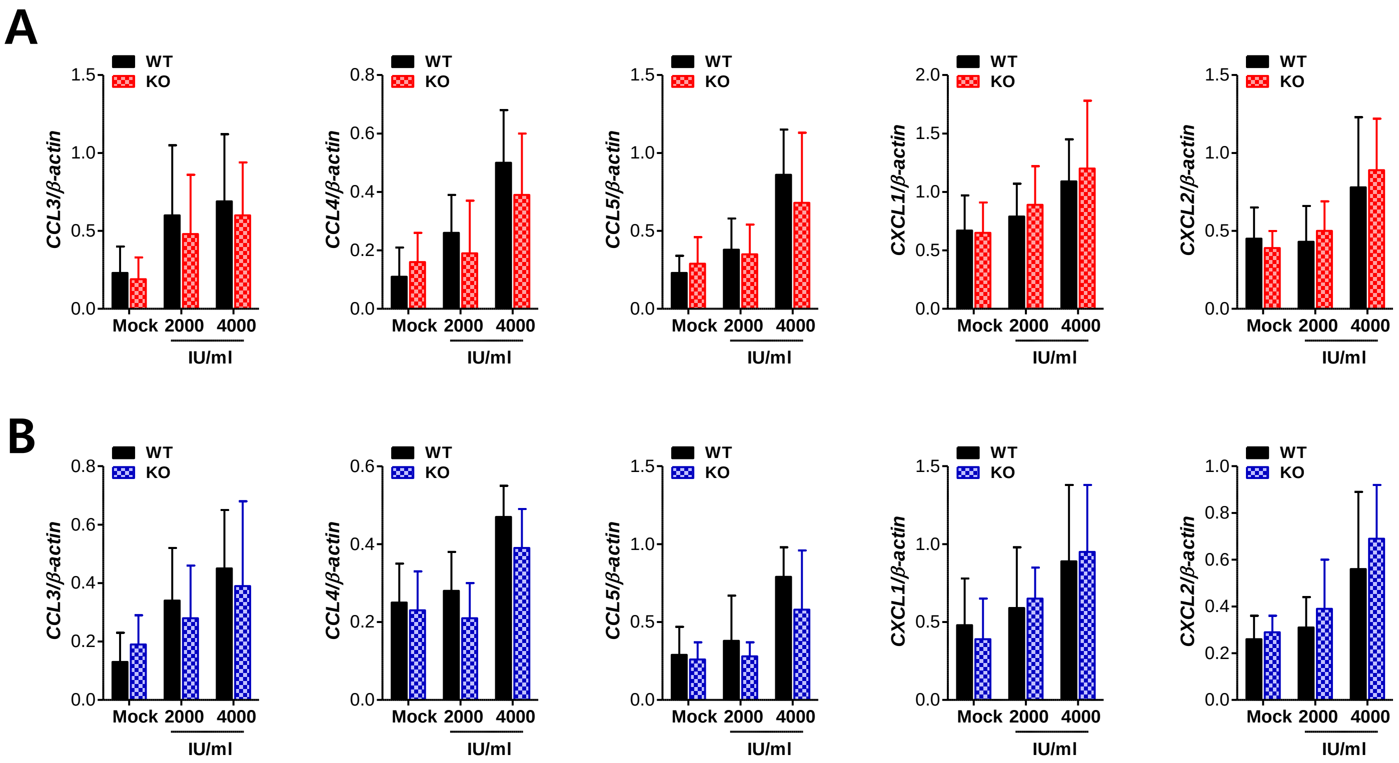

S5 Fig. The role of IFN-I signaling in the production of CC and CXC chemokines from CD11b^{hi}F4/80^{hi} macrophages and CD11c^{hi}EpCAM^{+} DCs. Vaginal CD11b^{hi}F4/80^{hi} macrophages (A) and CD11c^{hi}EpCAM^{+} DCs (B) isolated from WT and *IFNAR* KO mice were stimulated with recombinant IFN-α (2,000 and 4,000 IU/ml) for 6 h. The expression of CC and CXC chemokines were determined by real-time qRT-PCR. Data represent the average ± SD derived from three individual experiments (*n*=4-5).
