# Supplementary material for: Distinct Upstream Role of Type I IFN Signaling in Hematopoietic Stem Cell-Derived and Epithelial Resident Cells for Concerted Recruitment of Ly-6Chi Monocytes and NK Cells via CCL2-CCL3 Cascade
Source: PLoS Pathog. 2015 Nov 30;11(11):e1005256. doi: 10.1371/journal.ppat.1005256 (PMC4664252; doi:10.1371/journal.ppat.1005256)
Supplement: S6 Fig — (A and B) BL/6 mice were treated with clodronate liposomes via both intravenous (i.v.) and i.vag. routes. Resident CD11bhiF4/80hi macrophages in vaginal tract were determined by flow cytometric analysis 24 h after clodronate treatment. (C and D) CD11c-DTR Tg mice were administered DT via both intraperitoneal (i.p.) and i.vag. routes. Resident CD11chi DCs were determined by flow cytometric analysis 24 h after DT treatment. Values in the representative dot-plots denote the average percentages of vaginal macrophages and DCs derived from at least four independent samples, and data in the bar chart represent the average ± SD derived from three individual experiments (n = 4–5). **, p<0.01; ***, p<0.001 compared with the levels of the indicated group. (PDF) [file ppat.1005256.s006.pdf]

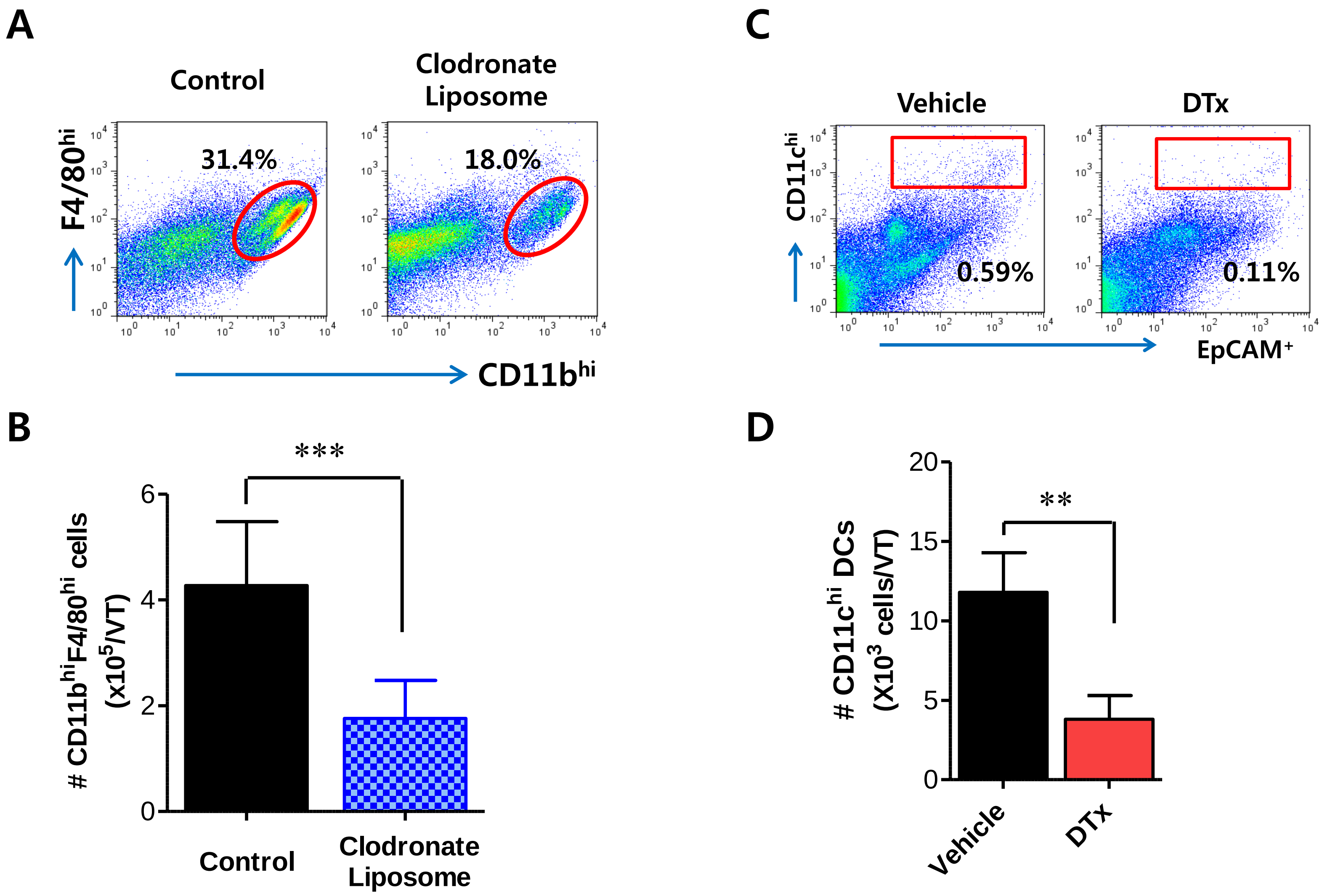

S6 Fig. Depletion of CD11b^{hi}F4/80^{hi} macrophages and CD11c^{hi} DCs. (A and B) BL/6 mice were treated with clodronate liposomes via both intravenous (i.v.) and i.vag. routes. Resident CD11b^{hi}F4/80^{hi} macrophages in vaginal tract were determined by flow cytometric analysis 24 h after clodronate treatment. (C and D) CD11c-DTR Tg mice were administered DT via both intraperitoneal (i.p.) and i.vag. routes. Resident CD11c^{hi} DCs were determined by flow cytometric analysis 24 h after DT treatment. Values in the representative dot-plots denote the average percentages of vaginal macrophages and DCs derived from at least four independent samples, and data in the bar chart represent the average ± SD derived from three individual experiments (*n*=4-5). **, $p<0.01$; ***, $p<0.001$ compared with the levels of the indicated group.
