# Supplementary material for: Distinct Upstream Role of Type I IFN Signaling in Hematopoietic Stem Cell-Derived and Epithelial Resident Cells for Concerted Recruitment of Ly-6Chi Monocytes and NK Cells via CCL2-CCL3 Cascade
Source: PLoS Pathog. 2015 Nov 30;11(11):e1005256. doi: 10.1371/journal.ppat.1005256 (PMC4664252; doi:10.1371/journal.ppat.1005256)
Supplement: S7 Fig — BL/6 mice were treated with GdCl3 via both i.v. and i.vag. routes, and were infected i.vag. with HSV-1 24 h after GdCl3 treatment. (A) Selective reduction of CD11b+Ly-6Chi monocyte infiltration by GdCl3-mediated inhibition of resident CD11bhiF4/80hi macrophages. Infiltrated CD11b+Ly-6Chi monocytes were determined by flow cytometric analysis at 24 h pi. (B) Reduced recruitment of NK cells in GdCl3-treated mice. CD3−NK1.1+DX5+ NK cells in vaginal tract were analyzed in GdCl3-treated mice at 48 h pi. (C) Viral replication in vaginal tract of GdCl3-treated mice. Viral titers in vaginal lavages of GdCl3-treated mice were determined by plaque assay at 6, 12, and 24 h pi. Values in the representative dot-plots denote the average percentages of Ly-6Chi monocytes, Ly-6Ghi granulocytes and NK cells derived from at least four independent samples, and data in the bar chart represent the average ± SD derived from three individual experiments (n = 4–5). **, p<0.01; ***, p<0.001 compared with the level of the indicated group. (PDF) [file ppat.1005256.s007.pdf]

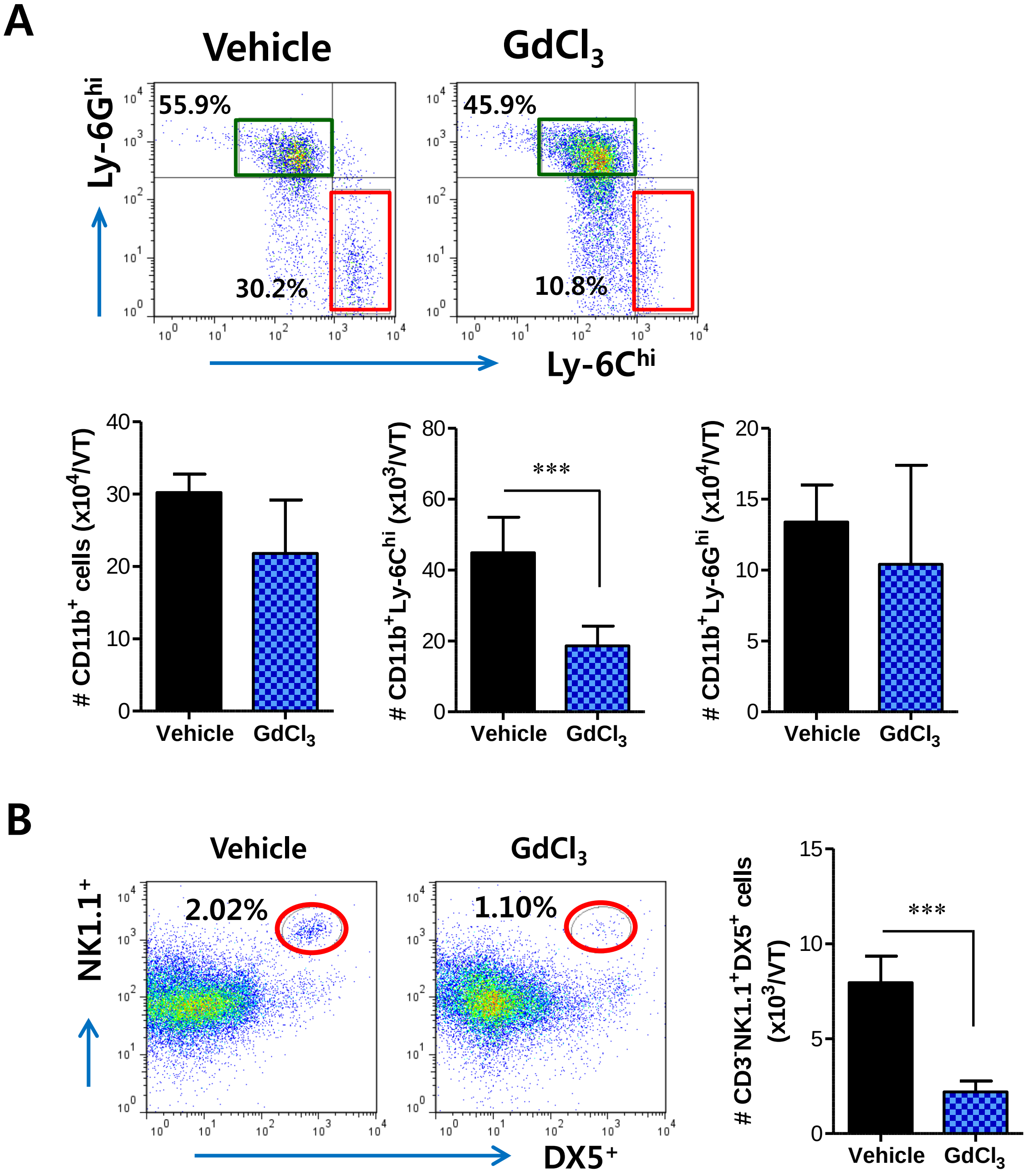

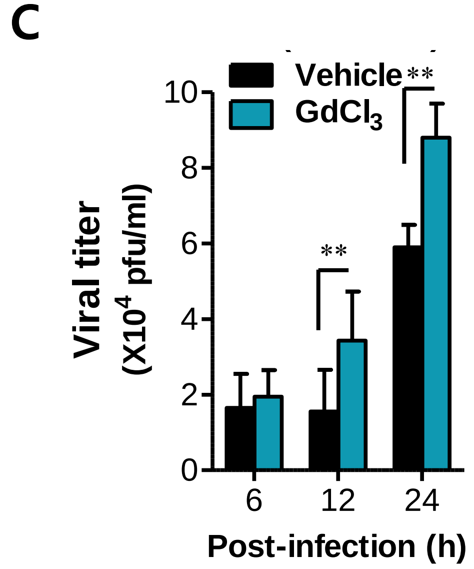

S7 Fig. Selective inhibition of resident CD11b^{hi}F4/80^{hi} macrophages by $GdCl_3$ reduces early infiltration of CD11b^{+}Ly-6C^{hi} monocytes and NK cells. BL/6 mice were treated with $GdCl_3$ via both i.v. and i.vag. routes, and were infected i.vag. with HSV-1 24 h after $GdCl_3$ treatment. (A) Selective reduction of CD11b^{+}Ly-6C^{hi} monocyte infiltration by $GdCl_3$-mediated inhibition of resident CD11b^{hi}F4/80^{hi} macrophages. Infiltrated CD11b^{+}Ly-6C^{hi} monocytes were determined by flow cytometric analysis at 24 h pi. (B) Reduced recruitment of NK cells in $GdCl_3$-treated mice. CD3^{-}NK1.1^{+}DX5^{+} NK cells in vaginal tract were analyzed in $GdCl_3$-treated mice at 48 h pi. (C) Viral replication in vaginal tract of $GdCl_3$-treated mice. Viral titers in vaginal lavages of $GdCl_3$-treated mice were determined by plaque assay at 6, 12, and 24 h pi. Values in the representative dot-plots denote the average percentages of Ly-6C^{hi} monocytes, Ly-6G^{hi} granulocytes and NK cells derived from at least four independent samples, and data in the bar chart represent the average ± SD derived from three individual experiments (n=4-5). **, $p<0.01$; ***, $p<0.001$ compared with the level of the indicated group.
