# Supplementary material for: Distinct Upstream Role of Type I IFN Signaling in Hematopoietic Stem Cell-Derived and Epithelial Resident Cells for Concerted Recruitment of Ly-6Chi Monocytes and NK Cells via CCL2-CCL3 Cascade
Source: PLoS Pathog. 2015 Nov 30;11(11):e1005256. doi: 10.1371/journal.ppat.1005256 (PMC4664252; doi:10.1371/journal.ppat.1005256)
Supplement: S1 Table — (PDF) [file ppat.1005256.s008.pdf]

S1 Table. Specific primers for PCR amplification of cytokines and chemokines.

Target gene		Primer sequence (5’-3’)[b]	Position cDNA	Gene Bank ID
**TNF-α**	F	CGT CGT AGC AAA CCA CCA AG	438-457	NM_013693
	R	TTG AAG AGA ACC TGG GAG TAG ACA	564-587	
**IL-1β**	F	TCC AAG AAA GGA CGA ACA TTC G	535-559	NM_008361
	R	TGA GGA CAT CTC CCA CGT CAA	679-700	
**IL-6**	F	TGG GAA ATC GTG GAA ATG AG	209-228	NM_031168
	R	CTC TGA AGG ACT CTG GCT TTG	442-462	
**IL-10**	F	CAA CAT ACT GCT AAC CGA CTC CT	1-24	NM_010548.1
	R	TGA GGG TCT TCA GCT TCT CAC	24-44	
**TGF- β**	F	GTG TGG AGC AAC ATG TGG AAC TCT	1-24	NM_011577.1
	R	TTG GTT CAG CCA CTG CCG TA	25-44	
**IL-15**	F	ACA TCC TAC TCG TGC TAC TTG TG	106-121	NM_008357.1
	R	CAT TGC AGT AAC TTT GCA ACT GG	399-419	
**IFN- α**	F	TGT CTG ATG CAG CAG GTG G	367-385	NM_008334.3
	R	AAG ACA GGG CTC TCC AGA C	514-532	
**IFN- β**	F	TCC AAG AAA GGA CGA ACA TTC G	106-121	NM_010510
	R	TGA GGA CAT CTC CCA CGT CAA	399-419	
**IFN-γ**	F	CAG CAA CAA CAT AAG CGT CA	119-220	NM_008337.3
	R	CCT CAA ACT TGG CAA TAC TCA		
**CCL2**	F	AAA AAC CTG GAT CGG AAC CAA	347-367	NM_011333
	R	CGG GTC AAC TTC ACA TTC AAA G	426-447	
**CCL3**	F	CCA AGT CTT CTC AGC GCC AT	158-177	NM_011337.2
	R	GAA TCT TCC GGC TGT AGG AGA AG	206-228	
**CCL4**	F	TTC TGT GCT CCA GGG TTC TC	128-147	NM_013652.2
	R	GAG GAG GCC TCT CCT GAA GT	388-407	
**CCL5**	F	CCC TCA CCA TCA TCC TCA CT	77-96	NM_009917
	R	CTT CTT CTC TGG GTT GGC AC	275-294	
**CXCL1**	F	CGC TGC TGC TGC TGG CCA CC	158-177	NM_008176.2
	R	GGC TAT GAC TTC GGT TTG GG	206-228	
**CXCL2**	F	ATC CAG AGC TTG AGT GTG ACG C	77-96	NM_009140.2
	R	AAG GCA AAC TTT TTG ACC GCC	275-294	
**CXCL10**	F	AAG TGC TGC CGT CAT TTT CT	347-367	NM_021274.1
	R	CAT TCT TTT TCA TCG TGG CA	426-447	
**β -actin**	F	TGG AAT CCC TGT GGG ACC ATG AAA C	128-147	NM_007393.3
	R	TAA AAC GCA GCT CAG TAA CAG TCC G	388-407	

[a]IL, interleukin; TNF-α, tumor necrosis factor- α; IFN, interferon; CCL, (C-C motif) ligand; CXCL, (C-X-C motif) ligand

[b]F, forward primer; R, reverse primer
